# Sex Differentiation and Long‐Distance Gene Flow in the Elusive Antarctic Fish *Aethotaxis mitopteryx*

**DOI:** 10.1002/ece3.71847

**Published:** 2025-08-20

**Authors:** Luca Schiavon, Thomas Desvignes, Fabrizia Ronco, Michael Matschiner, Martina Gastaldi, Thore Koppetsch, Audun Schrøder‐Nielsen, John H. Postlethwait, Alessia Prestanti, Federica Stranci, Santiago G. Ceballos, Felix C. Mark, Magnus Lucassen, Emilio Riginella, Mario La Mesa, Chiara Papetti

**Affiliations:** ^1^ Biology Department University of Padova Padova Italy; ^2^ Institute of Neuroscience University of Oregon Eugene Oregon USA; ^3^ Department of Biology University of Alabama at Birmingham Birmingham Alabama USA; ^4^ Natural History Museum University of Oslo Oslo Norway; ^5^ Department of Environmental Sciences University of Basel Basel Switzerland; ^6^ Instituto de Ciencias Polares, Ambiente y Recursos Naturales (ICPA), Universidad Nacional de Tierra del Fuego (UNTDF) Ushuaia Argentina; ^7^ Alfred Wegener Institute Helmholtz Centre for Polar and Marine Research Bremerhaven Germany; ^8^ Zoological Station Anton Dohrn Napoli Italy; ^9^ CNR‐ISP Institute of Polar Sciences Bologna Italy; ^10^ National Biodiversity Future Centre Palermo Italy

**Keywords:** *Aethotaxis mitopteryx*, *Dissostichus* spp., Notothenioidei, RAD‐sequencing, sex‐chromosomes, Southern Ocean

## Abstract

Understanding population connectivity in the marine realm is crucial for conserving biodiversity, managing fisheries, and predicting species responses to environmental change. This is particularly important in Antarctic waters, where unique evolutionary histories and extreme conditions shape marine biodiversity. The longfin icedevil *Aethotaxis mitopteryx* is an elusive notothenioid fish endemic to Antarctic waters. To explore population connectivity in *A. mitopteryx* , we used RAD‐seq to investigate the genetic differentiation of two populations, one from the Eastern Weddell Sea and the other from the Eastern Antarctic Peninsula, two regions of ecological relevance greatly impacted by climate change. Despite spatial separation, analyses revealed no significant genetic differentiation between the two populations, suggesting extensive gene flow. A pronounced genetic distinction was, however, observed between males and females. This differentiation was largely localized to a specific chromosome, implying a genetic sex determination system with males being the heterogametic sex. These findings contribute novel insights into the genetic structure of *A. mitopteryx* populations and expand our understanding of genetic mechanisms in Antarctic fish. This study provides a foundation for further investigations into the evolutionary and ecological implications of sex chromosome differentiation in extreme environments.

## Graphical abstract

This genetic study of a rare Antarctic fish, the longfin icedevil ( *Aethotaxis mitopteryx* ) investigates population connectivity across the Weddel Sea, revealing little to no differentiation even over vast geographic distances. Unexpectedly, the strongest genetic differentiation is instead observed between male and female fishes, which suggests a heterochromosome sex determination system in the longfin icedevil.

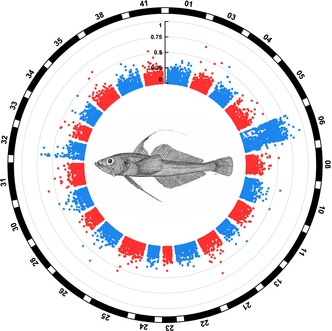

## 1 Introduction

Understanding population connectivity (i.e., the exchange of individuals among populations) and monitoring connectivity variability in the marine environment provide indications about a species' potential for population recovery and its capacity for re‐colonization and adaptation in case of demographic collapse (Hilário et al. 2015). In the marine environment, variation in levels of intraspecific connectivity typically reflects the interacting effect of different biological variables (e.g., natural and sexual selection, population size, pelagic larval duration, adult migratory behavior or dispersal capacity that might be different in males and females in case of sex‐biased dispersal, mating strategies, maximum adult size, egg type and other life history traits) and physical variables (e.g., physical barriers, currents, temperature, etc.) (Ritchie et al. 2007; Chopelet et al. 2008). Studies that address the biophysical mechanisms and the spatial and temporal scale of population connectivity are important not only to enhance our understanding of the ecology and evolutionary trajectories of marine organisms, but also to refine conservation and management strategies. In particular, studies of genetic connectivity (i.e., gene flow, population genetic structure) are useful to trace short‐ and long‐term variations in patterns of differentiation and genetic variability in response to environmental parameters, species‐specific life history traits, and human exploitation, which are essential data for the design of conservation measures. This is especially true for spatial approaches to resource management, such as initiatives adopted by the Commission for the Conservation of Antarctic Marine Living Resources (CCAMLR) for a network of marine protected areas (MPAs), including one already established in the Ross Sea (CCAMLR 2016) and in the South Orkney Islands (Trathan and Grant 2020) and others under evaluation, such as one proposal in the Weddell Sea (Teschke et al. 2021) and in the Western Antarctic Peninsula (CCAMLR 2020). Ideally, decisions governing the choice of size, number, spacing, and location of protected areas reflect species‐specific patterns of population connectivity, dispersal, and use of habitat, gene flow, and genetic structure (Boscari et al. 2019; Halpern 2003).

In this context, Antarctic fish of the suborder Notothenioidei can serve as models for population connectivity because their distinct ecologies, life history traits, and the environmental and hydrographic context of the Southern Ocean provide insights into diverse scales of connectivity and imply different impacts of biophysical factors.

Notothenioidei account for over 70% of the fish species present in marine communities in the Southern Ocean over the Antarctic continental shelf, and for over 90% of the fish biomass (Eastman 2024). They are considered one of the best examples of a marine species flock (Eastman and McCune 2000; Lecointre et al. 2013) and have evolved a range of shared and some unique physiological adaptations and life history characteristics. Well‐known adaptations to cold waters include the ability to synthesize antifreeze glycoproteins (AFGP) and antifreeze‐potentiating proteins (AFPP) (DeVries and Wohlschlag 1969; Duman 2015).

Notothenioids lack swim bladders, but the majority of species show increased buoyancy that appears to facilitate benthopelagic and benthic lifestyles (Eastman 1985, 2017, 2024; Eastman and Sidell 2002). In a few notothenioid species, a combination of increased lipid deposition and reduced skeletal development allows further increased buoyancy up to the point of neutrality (Chen et al. 2019; Eastman 2005, 2024). The resulting change in lifestyle, sometimes referred to as pelagization, is shown most strongly by the subfamilies Pleuragramminae and Dissostichinae (family Nototheniidae, classification following Duhamel et al. 2014). The two subfamilies are sister lineages and diverged early within the clade of Antarctic notothenioids (Near et al. 2018). The subfamily Pleuragramminae includes the Antarctic silverfish ( *Pleuragramma antarcticum* ) as its sole extant representative, and Dissostichinae includes the Antarctic and Patagonian toothfishes ( *Dissostichus mawsoni* and *D. eleginoides* ) and the longfin icedevil ( *Aethotaxis mitopteryx* )—all of which show adaptations towards neutral buoyancy (Eastman and DeVries 1989 for *P. antarcticum* ; Near et al. 2003 for *D. mawsoni* ; Eastman 1993 for *D. eleginoides* ; Near et al. 2007 for *A. mitopteryx* ). Two other species assigned to Dissostichinae, *Gvozdarus svetovidovi* and *G. balushkini* (Duhamel et al. 2014), are presumed to be pelagic, but they are extremely rare, and our knowledge of their biology is based on no more than three specimens (Eastman and Voskoboinikova 2024).

Many species of the Nototheniidae family are characterized by long pelagic larval phases (Kellermann 1990). These extended pelagic stages are exposed to the features of physical circulation of the Southern Ocean that connect also very distant sites. The most prominent feature of the Southern Ocean circulation is the Antarctic Circumpolar Current (ACC), which flows clockwise around the Antarctic continent. In proximity to the Western Antarctic Peninsula, the ACC moves over the slope and continues eastward and seaward, offshore of the South Shetland Islands (Ryan et al. 2016). In contrast to the ACC, the Antarctic Slope Current (ASC) and the Antarctic Coastal Current (AACC) flow westward and closer to the coast (Graham et al. 2013; Thompson et al. 2018). Flowing in opposite directions, the ACC and the ASC contribute to the northern and southern boundaries, respectively, of the Weddell Gyre, a mainly wind‐driven, cyclonic ocean gyre (Ryan et al. 2016).

Since species so far examined have pelagic early life stages that last over several months and often more than a year (Kock 2005 and La Mesa and Ashford 2007, 2008 for the Channichthyinae subfamily and Loeb et al. 1993 for Nototheniidae in general), notothenioids have traditionally been assumed to have high dispersal potential driven by the main circulation features, like the ACC (see Matschiner et al. 2015). Dispersal potential over long distances is often associated with expectations of high gene flow and lack of population structure (Hauser and Carvalho 2008). This conclusion has been confirmed in studies on some benthic species (Damerau et al. 2012; Jones et al. 2008; Matschiner et al. 2009; Papetti et al. 2012) but for some species, different levels of population structure have been described (Bernal‐Durán et al. 2024; Damerau et al. 2014; Papetti et al. 2009; Schiavon et al. 2023; Van de Putte et al. 2012). Among pelagic species, two have been particularly well studied: *D. mawsoni*, because of its high commercial value (Hanchet et al. 2015), and *P. antarcticum* , because of its high abundance and its key role in the Antarctic food web (La Mesa and Eastman 2012; Vacchi et al. 2017). For *D. mawsoni* , no evidence of genetic structure was found at the circum‐Antarctic level (Ceballos et al. 2021; Maschette et al. 2023). However, the pelagic and circumpolar *P. antarcticum* showed strong gene flow on large spatial scales but reductions in gene flow linked to discontinuities in the major current systems flowing along the continental shelf and slope (Caccavo et al. 2018).

Given these premises, we therefore hypothesized that *A. mitopteryx* also does not show signs of population structure. In fact, its ability for active swimming and its pelagic lifestyle, which expose it to current bidirectional transport with ACC, ASC, and AACC, especially during the larval phase, likely ensures a high dispersal capacity over large distances and thus high connectivity between areas. The absence of evidence for geographical population structure, however, would not exclude other types of population genetic structures based on age‐cohort division, sex (Benestan et al. 2017), sex‐specific dispersal (Sandoval Laurrabaquio‐A et al. 2019), mating types (Sonsthagen et al. 2012) or local adaptation (Benestan et al. 2016).

So far, though, *A. mitopteryx* connectivity patterns are unknown because of the species' elusive nature and the limited number of specimens sampled and studied to date. Indeed, *A. mitopteryx* is one of the least studied species among notothenioids. Although its presence has been recorded all around the Antarctic continent (DeWitt et al. 1990; Zimmermann 1997), its distribution might be patchy because catches have been scarce and irregular or because the fishing gears used are not specific for this pelagic fish. Kunzmann and Zimmermann (1992) reported that the four expeditions of the German research vessel (R/V) *Polarstern* carried out between 1985 and 1992 in the Weddell and Lazarev Seas yielded a total of 99 *A. mitopteryx* , representing 90% of the total catches of this species reported until then. During two more recent *Polarstern* expeditions carried out over a large area of the eastern and south‐eastern Weddell Sea in 2013–2014 (PS82) and 2015–2016 (PS96), a few specimens of *A. mitopteryx* were caught at two stations north of Cape Vestkapp (in Queen Maud Land) out of a total of 56 stations, confirming the rarity of observations of this species. During these two expeditions, the species was found along the continental slope at depths greater than 700 m (La Mesa et al. 2018) near the average position of the Antarctic Slope Current (ASC; Thompson et al. 2018), implying exposure to the along‐shelf circulation. Similarly, to the best of our knowledge, only ten specimens were caught on the Antarctic Peninsula at depths greater than 600 m during the *Polarstern* expedition in 2018 (PS112).

Despite being rarely captured, *A. mitopteryx* is considered to have a higher reproductive potential than most notothenioids because it is assumed to be the longest‐lived notothenioid species (up to 36 years for males and 62 years for females) and because it reproduces for the first time at 30%–50% of its maximum age (around 11 and 32 years for males and females, respectively; La Mesa et al. 2018). In contrast, all other notothenioids for which we have estimates reach sexual maturity at 50%–80% of their life span (La Mesa and Vacchi 2001). Unlike many notothenioid species, *A. mitopteryx* also has a pronounced sexual dimorphism in size and life expectancy, with females being larger and longer‐lived than males (La Mesa et al. 2018). Despite the limited amount of samples, the striking sexual dimorphism in size and life span found in *A. mitopteryx* can be considered quite reliable, being based on female and male samples of similar size as reported in La Mesa et al. (2018). Moreover, a few other notothenioid species also show sexual dimorphism in size and life expectancy (La Mesa et al. 2021); however, in those cases, the dimorphism is usually associated with differences in body colouration and fin morphology (Kock and Kellermann 1991). However, we cannot exclude that males and females have different behaviors or occupy different areas or depths and that these are therefore caught with different frequencies, thus biasing the results. Unfortunately, due to the complicated logistics of sampling in the Southern Ocean and of coordinating among and obtaining samples from different institutes and countries doing research in Antarctica, we cannot test this hypothesis yet.

Sexual dimorphism in fish, where males and females differ in size or life span, can significantly impact recruitment and demography by influencing factors like mating success, reproductive output, and growth rates. Indeed, in most gonochoristic species, size is a key factor in mate choice and sexual competition, favoring larger males in the case of sperm competition and larger females that are able to produce more eggs per season. Sexual dimorphism in growth rates directly influences longevity, which in turn determines the timing of sexual maturity and recruitment rates, having the potential to alter the population demographic dynamics. Size differences between sexes can also play a role in how fish populations interact within their environment, leading to different resource utilization and, ultimately, to food niche and spatial segregation, which might negatively affect mating success and the number of recruits determining the strength of connectivity (Prchalová et al. 2022; Ritchie et al. 2007).

These observations on life history traits suggest that for *A. mitopteryx* , the impact of differential recruitment and demographic fluctuations over time could play a possibly stronger role in shaping connectivity than in other Pleuragramminae and Dissostichinae. Hence, an assessment of *A. mitopteryx* population genetic structure requires the integration of biological and physical mechanisms to explain connectivity, including aspects of sex differentiation.

Therefore, in this study, we conducted the first assessment of population structure in *A. mitopteryx* between the two opposite sides of the Weddell Sea—the East Antarctic Peninsula and the East Weddell Sea (separated by ~1900 km in a straight line). These two locations are of great ecological relevance. On one hand, the Antarctic Peninsula is experiencing one of the fastest warming rates of the planet. On the other hand, the Weddell Sea was considered one of the last regions of the Southern Ocean to experience the consequences of climate change due to its extensive and persistent sea‐ice cover and ocean currents pattern (Teschke et al. 2021). However, recent studies have reported that the Weddell Sea has also seen dramatic changes, particularly in sea ice cover since 2016 (Raphael et al. 2025; Turner et al. 2020). It has also experienced significant long‐term warming, particularly in deep waters (Strass et al. 2020).

Moreover, since our analysis gave us unexpected access to genetic data differentiating sexes, in this study, we were able for the first time to investigate the genetic bases of sex differentiation in *A. mitopteryx* .

Given the difficulty of gathering samples from the Southern Ocean and especially for *A. mitopteryx* , which limits the sample size, this study nonetheless represents a unique opportunity to provide a first assessment of connectivity and genetic sex differentiation in this little‐studied notothenioid, one of the few pelagic species and the longest‐lived notothenioid known so far.

## 2 Materials and Methods

### 2.1 Samples, RAD‐Seq Library and DNA Sequencing

**FIGURE 1:**
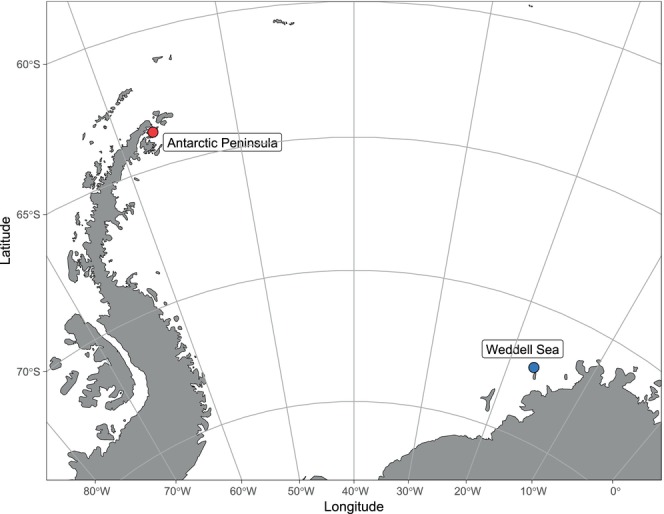
Map of the sampling sites of the two populations of *Aethotaxis mitopteryx* analyzed in this study. Coordinates of the two sampling sites are 63°42′02.7″S 57°02′01.0″W for EAP and 72°17′47.4″S 16°50′49.8″W for EWS. Map drawn with R package ggOceanMaps (Vihtakari 2024) and manually modified.

We analyzed 10 *A. mitopteryx* specimens (4 males and 6 females) collected from the East Weddell Sea (EWS) during PS96 in January 2016 and eight specimens (2 males and 6 females) collected from the Antarctic Peninsula sector of the Weddell Sea during PS112 in April 2018 (hereafter East Antarctic Peninsula, EAP, Figure 1). The two sampling sites are separated by approximately 1900 km in a straight line. All samples were collected by bottom trawl, at depths of 878 m in EWS and at 641 m in EAP. The sex of each specimen was first identified by visual inspection of the gonads at dissection and later confirmed by gonad histology for some specimens as previously described for other notothenioid species (e.g., Riginella et al. 2021). DNA was extracted and restriction site associated DNA sequencing (RAD‐seq; Baird et al. 2008) libraries were prepared following Schiavon et al. (2024). Briefly, DNA was digested with a single restriction enzyme –*SbfI*– and the first indexed adapter (P1) was ligated to the cut end. All samples were then pooled (multiplexed) and sheared using Covaris. Fragments of the target length (between 400 and 600 bp) were isolated using a size selection step by gel electrophoresis. A second adapter (P2) was ligated to the free ends. Fragments ligated to both adapters, P1 and P2, were selectively enriched by PCR amplification before sequencing. Paired‐end sequencing with a read length of 150 bp was conducted on the Illumina HiSeq4000 platform at the Genomics & Cell Characterization Core Facility (GC3F, University of Oregon, USA).

### 2.2 Whole Genome Sequencing and de Novo Assembly

One *A. mitopteryx* female specimen (sampled in the East Weddell Sea) was selected to generate a *de novo* draft genome assembly following a dual‐sequencing strategy that combined short‐ and long‐read sequencing. Although the goal was to produce a hybrid assembly leveraging both data types, the process was ultimately shaped by limitations in long‐read sequencing coverage.

After DNA extraction (see above), we initially generated 250 bp paired‐end (PE) reads on an Illumina NovaSeq 6000 platform (KAPA Hyper prep PCA free library). Library preparation and sequencing were performed at the D‐BSSE Genomics Facility Basel of ETH Zurich (https://bsse.ethz.ch/genomicsbasel), yielding approximately 100‐fold sequencing depth. Remaining genomic DNA from multiple extracts was pooled and concentrated in a Speed‐Vac centrifuge to a total volume of 60 μL, size‐selected using a Blue Pippin electrophoresis platform (selecting molecules longer than 10 kb on a high pass plus cassette). Library preparation was performed using the SQK‐RAD004 kit followed by sequencing on an Oxford Nanopore Technologies MinION Mk1C device using an R9.4 flow cell. Unfortunately, the long‐read sequencing run resulted in only ~4‐fold genome coverage. Given this constraint, we tested multiple hybrid assembly strategies to make the best use of both data types. The most complete and contiguous draft genome assembly—judged by metrics such as N50, total assembly size, number of contigs, and BUSCO completeness—was obtained with the following assembly pipeline. First, Illumina short reads were trimmed for adapter sequences and low‐quality bases using trimmomatic ver. 0.39 (Bolger et al. 2014). Next, overlapping paired‐end reads were merged into longer reads using flash2 ver. 2.2.0 (Magoč and Salzberg 2011). The non‐overlapping paired‐end reads (~50%) were then assembled *de novo* using abyss ver. 2.3.1 (Jackman et al. 2017) with k‐mer size (k) set to 95 and minimum coverage cutoff (kc) set to 4. The resulting contigs were scaffolded with links ver. 1.8.6 (Warren et al. 2015), followed by patching with low‐coverage MinION long reads, and polished with merged Illumina reads, all using racon ver. 1.4.7 (Vaser et al. 2017). Continuity and completeness of the assembly were assessed using quast ver. 5.0.2 (Gurevich et al. 2013) and busco ver. 5.1.2 (Simão et al. 2015), respectively.

Given that the generated *A. mitopteryx* genome assembly remained fragmented, all scaffolds were ordered and oriented into larger scaffolds with the software ragtag ver. 2.1.0 (Alonge et al. 2022) and the option *scaffold* and the aligner unimap (https://github.com/lh3/unimap) using as a guide the closely related species *D. mawsoni* genome assembly (Lee et al. 2021; NCBI accession number: GCA_011823955.1). This step does not alter the original sequence of the *A. mitopteryx* genome assembly, but allows visualization of genetic variability at a larger scale. Hereafter, we will thus refer to *A. mitopteryx* scaffolds following the scaffold numeration in the *D. mawsoni* genome assembly.

Before submission to NCBI, the final *de novo* not‐scaffolded assembly was decontaminated using the ncbi foreign contamination screen (fcs) tool ver. 0.4.0 (Astashyn et al. 2024).

### 2.3 Bioinformatic Processing of RAD‐Seq Raw Reads, SNP Calling and Filtering

Raw RAD‐seq data were demultiplexed using the *process_radtags* module of stacks ver. 2.53 (Catchen et al. 2011; Rochette et al. 2019) and PCR duplicates were removed with the stacks tool *clone_filter*.

Illumina reads were then mapped to the newly generated and scaffolded *A. mitopteryx* genome assembly (see above) using bwa ver. 0.7.17 with default parameters (Li and Durbin 2009). SNP calling was performed with the stacks tools *gstacks* and *populations* with parameter –r 0.75 (minimum percentage of individuals required to process a locus) and –max‐obs‐het 0.75 (maximum observed heterozygosity allowed to process a SNP). The resulting VCF file was filtered with vcf tools ver. 0.1.17 (Danecek et al. 2011), retaining only genotypes with a minimum Phred‐scaled genotype quality of 30 and a sequencing depth between 5 and 40 (individuals failing these thresholds were marked as having a missing genotype). Only sites with mean sequencing depth between 10 and 40 were retained (sites failing these thresholds were removed from the data set). Singletons were removed. The R package SNPfiltR (DeRaad 2022; Knaus and Grünwald 2017) was used to convert heterozygous genotypes to missing data if they had an allele balance (i.e., ratio of reads showing the reference allele among all reads) outside the range 0.25–0.75, since they could derive from misaligned paralogs. SNPs with a missing data proportion above 0.15 were removed, and only SNPs at least 1000 bp apart were retained to obtain a thinned panel of unlinked markers.

### 2.4 Population Structure

The presence of population structure was first assessed using both a Principal Component Analysis (PCA) with the R package ‘adegenet’ ver. 2.1.10 (Jombart 2008) and unsupervised hierarchical clustering with admixture ver. 1.3.0 (Alexander et al. 2009). admixture was run to estimate individual ancestry proportions (Q) with cross‐validation, assuming a value of K from 1 to 5 for the estimation of the optimal number of clusters. To quantify differences between groups, the pairwise fixation index (*F*_ST_) as defined by Weir and Cockerham (1984) between sampling sites and sexes was calculated with the R package ‘StAMPP’ ver. 1.6.3 (Pembleton et al. 2013).

Haplotype‐based approaches can infer population structure at higher resolution and give a stronger signal of differentiation compared to SNP‐based methods because they exploit information from the full RAD haplotypes, which are generally more variable than individual sites (Bootsma et al. 2021; Leitwein et al. 2020). To implement this approach, we first discarded RAD loci with 10 or more SNPs from the stacks *populations* module output because such high SNP densities are likely to result from misaligned paralogous loci (https://www.milan‐malinsky.org/fineradstructure). Then, we ran *populations* with this subsample of loci to produce an input file for fineradstructure (Malinsky et al. 2018) and to calculate the divergence statistics φ_ST_ (a locus‐level version of *F*_ST_; Excoffier et al. 1992) and *D*_XY_ (absolute divergence; Nei 1987). fineradstructure computes a matrix of haplotype relationships between samples (the “co‐ancestry matrix”). φ_ST_ and *D*_XY_ were calculated comparing geographic regions (EAP vs. EWS) and between sexes. The computation was made per single locus and across a sliding window (specifying the—smooth option in *populations*, which implies a window size of 900 bp and the average weighted across the window according to a Gaussian distribution centered on the middle of the window).

### 2.5 Sex Differentiation

Since our analysis of population structure revealed a clear genetic differentiation between males and females (see Results), we conducted further analyses to investigate this aspect in *A. mitopteryx* .

Levels of homozygosity were calculated for all samples across the *A. mitopteryx* genome assembly to identify sex‐linked regions and the heterogametic sex. Homozygosity in sex‐linked regions in the heterogametic sex is expected to be much lower than that in sex‐linked regions in the homogametic sex and in non‐sex‐linked regions in both sexes. Individual levels of homozygosity per single scaffold were computed with vcftools.

If the sex chromosomes in the heterogametic sex are strongly differentiated or if the sex‐linked region is present in only the heterogametic sex, sequencing reads should differentially align in males and females, leading to an imbalance in sequencing depth along sex‐linked regions. To test for such a signal in *A. mitopteryx* , we followed the procedure described in Feller et al. (2021) and quantified sequencing depth per SNP separately for males and females with vcftools to then calculate female/male depth ratios. The ratios were corrected for a mean genome‐wide ratio to be centered at 1. Results were then averaged across a sliding window of 5 Mb with the function *winScan* in the R package ‘windowscanR’ (https://github.com/tavareshugo/WindowScanR).

Differences in the pattern of linkage disequilibrium (LD) could also indicate that recombination has been reduced or prevented between the differentiated sex chromosomes (McKinney et al. 2020) or, not exclusively, that strong selection has acted on sex‐linked genomic regions (Koch et al. 2022). LD was calculated with PLINK ver. 1.90 (Purcell et al. 2007). Results were averaged across genomic windows of 10 kb with ‘windowscanR’.

To identify sex‐linked markers, we used radsex ver. 1.2.0 (Feron et al. 2021). radsex analyzes raw sequencing reads rather than individual SNPs and identifies the presence or absence of each unique read (i.e., marker) in each individual. radsex then returns a list of markers that have a statistically different distribution between sexes. Following the software guidelines, we applied the command *process* to generate a table of marker depths for the whole dataset as well as the distribution of markers between males and females, using four different thresholds for minimum read depth in each specimen (1, 2, 5, 10). Then, markers significantly associated with sex were identified with the command *signif*. All markers were further aligned to the *A. mitopteryx* genome assembly to visualize their distribution and location along different scaffolds with the command *map*. Results were plotted with the R package ‘sgtr’ ver. 1.1.2 (Feron et al. 2021).

The scaffolds in the *D. mawsoni* genome assembly used to increase the contiguity of the *A. mitopteryx* assembly were not numbered following the chromosome naming convention established for notothenioid species (Bista et al. 2020, 2023; Kim et al. 2019), which follows the chromosome numbering in medaka, a species representative of the ancestral percomorph karyotype of 24 chromosomes (Kim et al. 2019). Thus, to facilitate comparison of our results with other species, we used the d‐ genies web application (Cabanettes and Klopp 2018) to align the original *D. mawsoni* genome assembly to the genome assembly of the more distantly related non‐Antarctic notothenioid *Cottoperca trigloides* (formerly known as *Cottoperca gobio* , NCBI accession number: GCF_900634415.1), in which chromosomes were numbered based on their orthology with medaka (Bista et al. 2020).

For comparative purposes, we repeated the same radsex procedure using *D. mawsoni* RAD‐seq data from Ceballos et al. (2021). We reanalyzed 12 males and 9 females for which the sex was confirmed by histological examination.

### 2.6 Search for Candidate Sex Determining Genes

Considering that many different sex determining genes have independently evolved in various fish lineages but often involved members of the conserved vertebrate sex‐determination and differentiation pathway, especially of the TGF‐β family (Adolfi, Herpin, and Schartl 2021; Kitano et al. 2024; Pan, Feron, et al. 2021), we searched for the presence and location of 35 putative teleost sex‐determining gene candidates (listed in Table S1) in the *A. mitopteryx* genome assembly using BLASTN.

## 3 Results

### 3.1 De Novo Genome Assembly

Our newly sequenced and *de novo* assembled *A. mitopteryx* genome was fragmented in 210,180 contigs, with a total length of 596 Mb and an N50 value of 9740 bp. The assembly contained 61.3% of the Actinopterygii BUSCO genes as complete sequences, while an additional 12.4% of the genes were present in fragmented form and 26.3% were missing from the assembly. More detailed statistics on genome continuity and completeness at different assembly stages are reported in Table S2. The scaffolding of the *A. mitopteryx* assembly on the *D. mawsoni* reference genome combined 10.6% of the contigs (corresponding to 51.6% of the total length) into 310 scaffolds. The unplaced contigs were joined into a single pseudoscaffold. Additional statistics related to assembly and scaffolding are reported in Tables S3 and S4.

### 3.2 Population Structure

**FIGURE 2:**
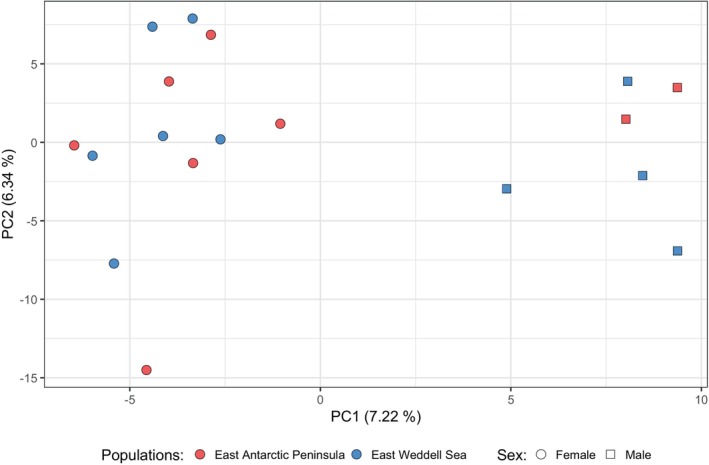
Scatter plot of the first two Principal Components across the SNP data set. Labels on the axes indicate the contributions of the displayed principal components to the variance.

The filtered VCF file included 10,335 SNPs. No geographic genetic structure was observed in the PCA plot, while males and females were separated along the first axis that explains 7.22% of the variance (Figure 2). Cross‐validation errors of admixture results indicated *K* = 1 as the optimal number of genetic clusters (Figure S1) and when plotting *K* = 2, individuals were not divided by sampling location or sex (Figure S2, top panel). Weir and Cockerham's (1984) *F*_ST_ between sampling sites was 0.00014 and not significant (*p*‐value = 0.431) while the *F*_ST_ between sexes was 0.02258 and significant (*p*‐value = 0.001).

The co‐ancestry matrix inferred by fineradstructure was consistent with the PCA and clustered the specimens according to their sex (Figure S3). When computing haplotype‐based divergence statistics between geographic locations, both φ_ST_ and *D*_XY_ were uniformly distributed along the scaffolds (Figure S4). However, φST was low on most of scaffold 06 compared to the rest of the assembly (Figure S4A). We conclude that evidence for genetic structure is lacking in these data for geographic sites but is readily evident for differences between sexes.

### 3.3 Sex Determination System

**FIGURE 3:**
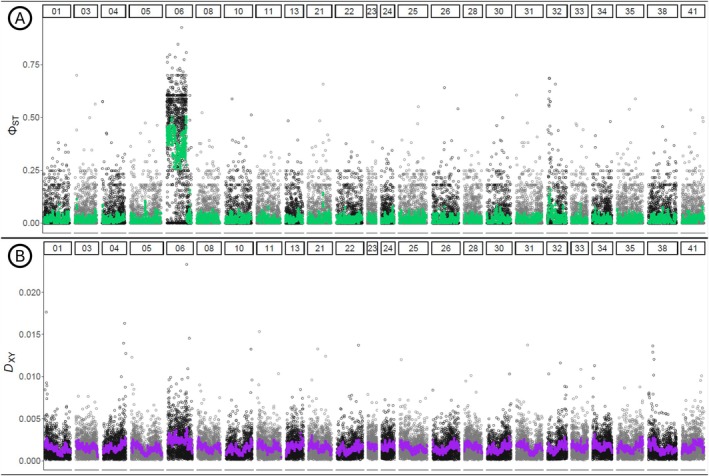
Manhattan plot of haplotype‐based divergence statistics φ_ST_ (A) and *D*_XY_ (B) calculated between males and females of *Aethotaxis mitopteryx* . Alternating black and gray dots represent single‐site values in different scaffolds. φ_ST_ smoothed average is displayed in green, *D*_XY_ smoothed average is displayed in purple.

When comparing sexes, φ_ST_ calculated based on the haplotypic frequencies of markers from scaffold 06 (39,811,704 bp long in the *D. mawsoni* assembly and 26,171,853 bp long in the scaffolded *A. mitopteryx* assembly) was much higher than φ_ST_ of all other scaffolds (Figure 3A). *D*_XY_, in contrast, was only slightly higher for scaffold 06 than for the other scaffolds (Figure 3B). For both statistics, the first ~80% of the scaffold (from 0 to ~21 Mb of the scaffold in the *A. mitopteryx* assembly) showed this sex‐linked pattern. When removing from the dataset all markers on scaffold 06 (SNPs and haplotypes), both PCA and fineradstructure (Figures S5 and S6) did not return any clustering, and the admixture result remained almost unchanged (Figure S2, bottom panel).

**FIGURE 4:**
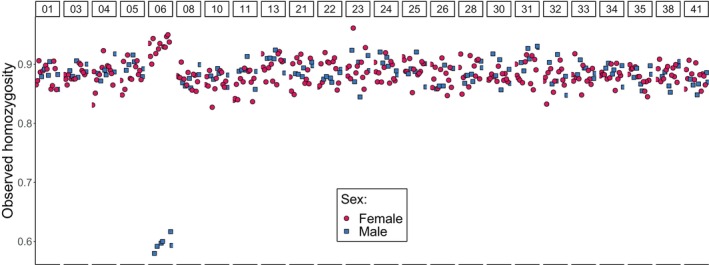
Observed homozygosity. Each dot represents an individual of *Aethotaxis mitopteryx* for each scaffold separately.

Homozygosity levels were also different between sexes on scaffold 06, but they were equivalent on all other scaffolds (Figure 4). On scaffolds other than scaffold 06, homozygosity of variable sites was about 90% and was similar between males and females (Figure 4). In stark contrast, on scaffold 06, males showed values around 60% homozygosity, which is much lower than the genome‐wide average of ~90%. In females, homozygosity values on scaffold 06 were slightly higher than the genome‐wide average (Figure 4). The higher heterozygosity in males compared to females suggests that males are the heterogametic sex.

**FIGURE 5:**
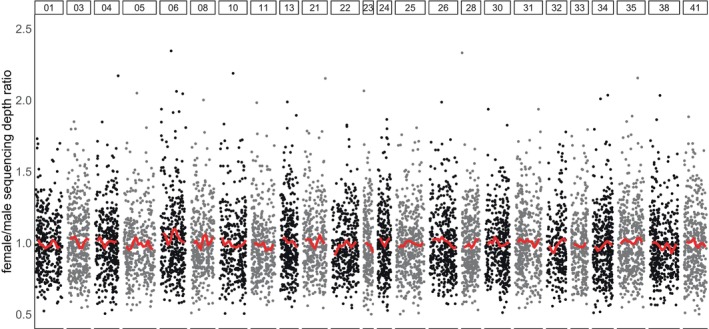
Per SNP sequencing depth ratio between females and males of *Aethotaxis mitopteryx* . Alternating black and gray dots represent single‐site values in different scaffolds. Smoothed average is displayed in red. All values were corrected for the mean ratio centered at 1.

Sequencing depth was similar between males and females along the entire genome assembly, without noticeable differences on scaffold 06 (Figure 5).

**FIGURE 6:**
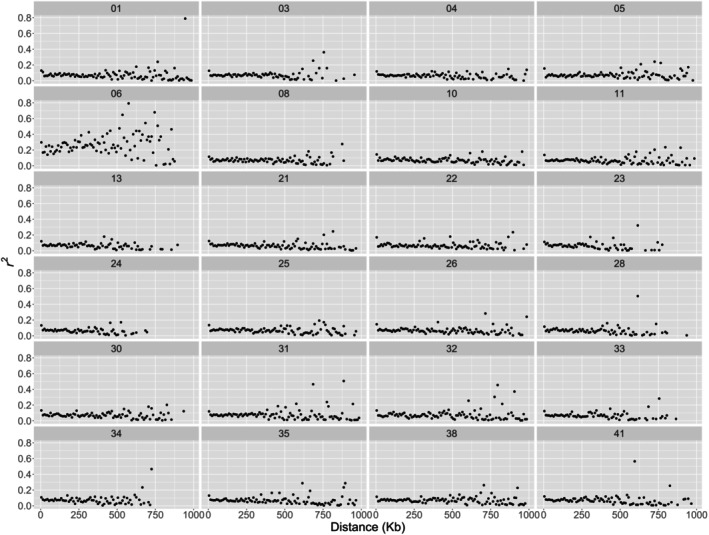
Linkage disequilibrium between SNPs along separate scaffolds considering all individuals of *Aethotaxis mitopteryx* analyzed in this study.

**FIGURE 7:**
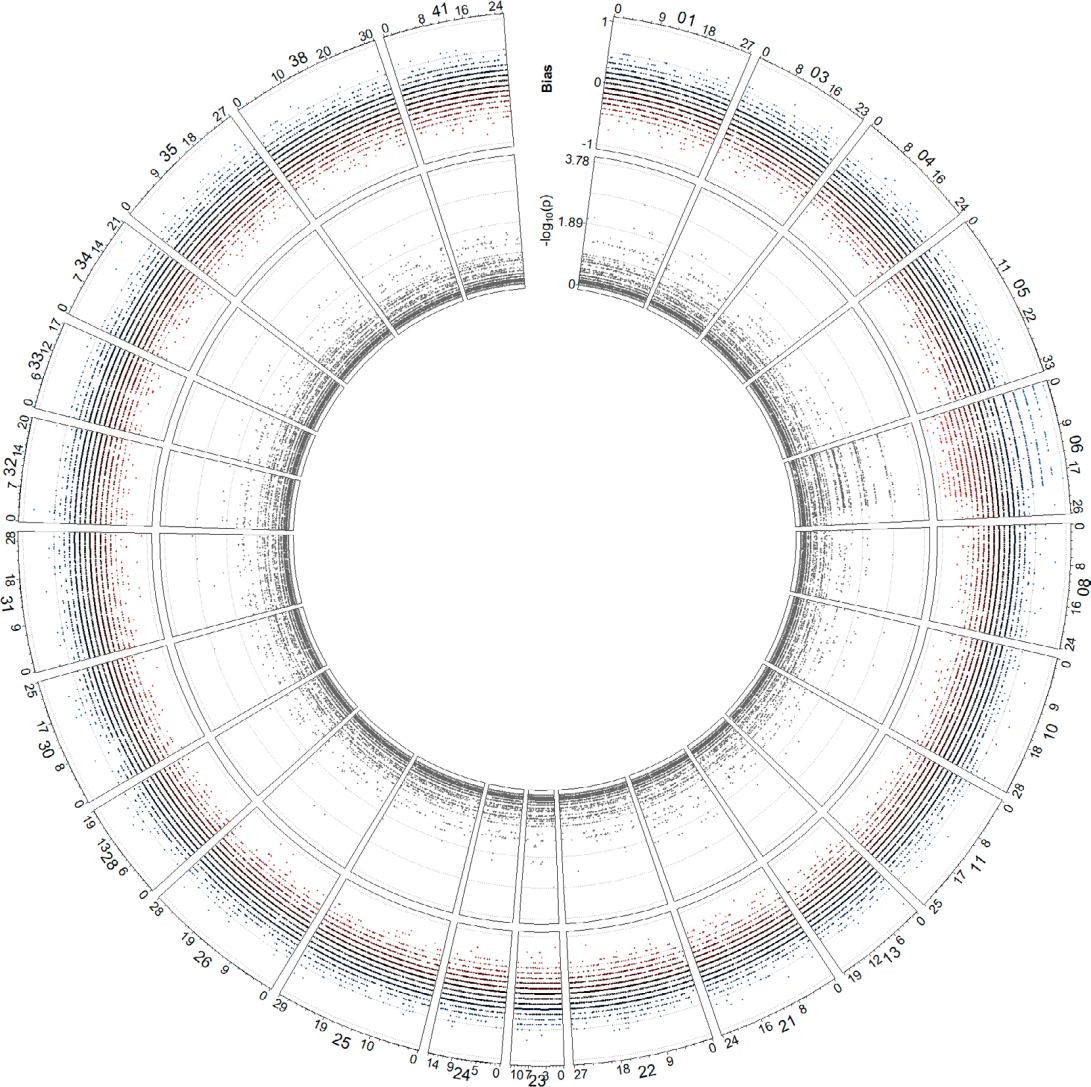
Plot showing the distribution of markers between females and males along the genome assembly, considering a sequencing depth threshold of 10. Each sector represents a different scaffold. The top track indicates the bias of a marker: If present in all males and in none of the females, a marker has a value of 1; if present in all females and in none of the males a marker has a value of −1. The bottom track indicates the probability of association with sex.

Linkage disequilibrium (LD) was higher in scaffold 06 than in the rest of the genome assembly (Figure 6). When analyzing males and females separately, no difference was found among scaffolds, although males showed overall higher LD values than females (Figure S7).

radsex recovered sex‐biased markers attributed exclusively to one or the other sex at all depth thresholds tested (Figure S8). Despite this result, none of the markers were significantly sex‐linked. However, when markers were mapped to the genome assembly using the radsex function *map*, a clear biased association between males and females was found on the first 20 Mb of scaffold 06 (Figure 7). When considering only markers on scaffold 06, more markers were male‐specific and almost none were female‐specific (Figure S9). Reducing the depth threshold for the radsex analysis consistently recovered more male‐specific markers and fewer female‐specific markers. Further, comparing the original *D. mawsoni* genome assembly to the *C. trigloides* genome assembly revealed that *D. mawsoni* scaffold 06 corresponds to *C. trigloides* chromosome 21 (Figure S10).

### 3.4 Sex Determining Genes

To identify sex determining gene candidates, we searched *A. mitopteryx* genome assembly for the genomic location of 35 genes known to be sex‐determining or involved in the sex differentiation pathway in other teleost species (Adolfi, Herpin, and Schartl 2021; Kitano et al. 2024; Pan, Kay, et al. 2021). None of the searched genes were located within the sex‐linked region on scaffold 06; however, a *bmpr2‐like* gene paralog was located on scaffold 06 of *A. mitopteryx* , 1.8 Mb upstream of the last sex‐linked marker (Table S1). A few other genes (*bmpr1bb*, *esr1*, *gsdf*, and *gata4*) were also located on scaffolds other than 06 that contained few isolated sex‐linked markers; however, in each case they were positioned several Mb away from the sex‐linked SNPs (Table S1).

### 3.5 Sex Differentiation in *Dissostichus mawsoni*

The reanalysis of *D. mawsoni* RAD‐seq data (Ceballos et al. 2021) using radsex did not reveal any sex‐linked marker at any studied depth thresholds (Figure S11) nor noticeable marker biases in any specific region of the genome assembly (Figure S12).

## 4 Discussion

In this study, we found high overall genomic homogeneity between two populations of *A. mitopteryx* sampled from the East Antarctic Peninsula (EAP) and the East Weddell Sea (EWS). In contrast, our analyses revealed a clear pattern of sex differentiation, which is based on sex‐biased markers concentrated along a single scaffold. High male heterozygosity suggests that *A. mitopteryx* displays a genetic sex determination system with males being the heterogametic sex. Thus, *A. mitopteryx* likely has a chromosomal sex‐determination system based on the presence of X and Y chromosomes.

### 4.1 Population Structure

None of the analyses we conducted to investigate population differentiation between the two sides of the Weddell Sea (i.e., PCA, unsupervised hierarchical clustering, and haplotype co‐ancestry matrix) detected any geographical population structure, with a low and statistically not significant genetic differentiation (*F*_ST_ ≈0.0001, *p*‐value = 0.431). Despite the large geographical distance between the two sampling sites, our result is consistent with the expectation that notothenioids have a high dispersal potential even on a continental scale, especially for pelagic species that may travel long distances as adults like Pleuragramminae and Dissostichinae. The long pelagic larval phase (from 2 months to up to 1 year depending on the species and locality; Kellerman 1989; North 2001), the local hydrography of different areas, and the dominant ocean currents in the Southern Ocean are generally considered to be the main drivers of population connectivity, often resulting in genetic homogeneity (Matschiner et al. 2015). In particular, the eastern and western sectors of the Weddell Sea, where our samples originated, may be connected by several currents, mainly the ACC, ASC, and AACC, that flow in different directions and at different depths (e.g., Figure 1 in Caccavo et al. 2018). It is also important to note that our two population samples were collected in different years (in 2016 for EWS and in 2018 for EAP). This could mean that gene flow is not only extensive across larger geographic distances but may also be sustained over time. The hypothesis that circulation promotes genetic connectivity between the two sides of the Weddell Sea was previously tested by Schiavon et al. (2021) for the icefishes of the genus *Chionodraco* (Nototheniidae, Channichthyinae), by combining Lagrangian particle tracking simulations with genetic analyses of nuclear (microsatellites) and mitochondrial DNA (D‐loop) markers. While Lagrangian simulations suggested limited bidirectional connectivity between the Western Antarctic Peninsula and the Eastern Weddell Sea, microsatellites and the D‐loop did not discriminate between *C. rastrospinosus* population samples collected in the EWS and in several other locations around the Antarctic Peninsula. Instead, the reanalysis of a subset of samples of Schiavon et al. (2021) using RAD‐seq revealed weak population differentiation (Schiavon et al. 2024). Taken together, the genetic results suggest that gene flow occurs, and several hypotheses explaining the discrepancy between Lagrangian simulations and genetic data were put forward. Connectivity may occur in a stepping‐stone fashion through intermediate locations between the two sides of the Weddell Sea, where particle release was not simulated because no specimen captures were ever reported from these areas, although their presence cannot be excluded (Kattner and Koch 2009). The movements of actively swimming adult individuals or the occasional strengthening of oceanographic currents due to weather conditions could also explain the connectivity between the two populations of *A. mitopteryx* . Since *A. mitopteryx* is a pelagic species, the hypothesis of connectivity driven also by adult movement may be particularly relevant. Indeed, studies on two other pelagic notothenioids, *P. antarcticum* and *D. mawsoni* , did not recover any signal of genetic differentiation at the circum‐Antarctic scale (Caccavo et al. 2018; Ceballos et al. 2021; Maschette et al. 2023). However, areas of *P. antarcticum* occurrence that displayed reduced gene flow were separated by discontinuities in the large‐scale ocean circulation, highlighting the importance of currents in along‐shelf population connectivity for pelagic species (Caccavo et al. 2018). In particular, populations from Charcot Island and Marguerite Bay, both below the Antarctic circle on the Western Antarctic Peninsula, showed genetic differentiation compared to nearby locations more north on the Western Antarctic Peninsula. Evidence that areas on opposite sides of the Antarctic continent are genetically homogeneous while closer locations host differentiated populations illustrates the importance of an extensive sampling design over space and time and suggests that environmental variability plays a key role in shaping population dynamics (Corso et al. 2022).

Our small sample size and spatial coverage certainly limited the power to detect subtle genetic differences hindering our capacity to provide conclusive evidence of population differentiation. In particular, while we focused mostly on aspects of neutral differentiation, we cannot exclude that the two populations differ at loci experiencing different selective pressures in the two geographic areas. Moreover, although we obtained genetic data that differentiate males and females (see next sections of the Discussion), the small sample size and limited availability of genetic data do not allow testing for sex‐biased dispersal (e.g., Prugnolle and de Meeus 2002). Therefore, although Antarctic field campaigns are logistically demanding, future studies should pay specific attention in accurately/appropriately sampling and preserving rare species such as *A. mitopteryx* and welcome international collaborations to expand sample sizes and spatial scale. New tools for species presence detection could also be used, like analysis of environmental DNA (eDNA, e.g., from water or sponge bycatch as in Jeunen et al. 2024). As a future perspective, analysis of whole‐genome sequences obtained via e.g., PoolSeq or low‐coverage Whole Genome Sequencing (lcWGS) and of a high‐quality reference genome assembly for *A. mitopteryx* should be combined with non‐genetic approaches to overcome the limits of small sample size. Indeed, otolith microchemistry and stable isotope analyses on individuals at different developmental stages and from various areas will also help verify that genetic connectivity is shaped by oceanographic currents.

### 4.2 Sex Differentiation and the Sex Determination System of *A. mitopteryx*

Males and females of *A. mitopteryx* are generally externally indistinguishable, although females grow larger than males (La Mesa et al. 2018). Our data, however, provide the first indication of genetic differences between sexes in *A. mitopteryx* . Both PCA and the co‐ancestry matrix computed using fineradstructure clearly showed the presence of two groups, perfectly separating males and females (*F*_ST_ ≈0.0226; *p*‐value = 0.001). In contrast, hierarchical clustering performed by admixture failed to identify the two groups. Although we cannot give a conclusive explanation for this difference, we hypothesize that it is due to the different assumptions the programs make about the data. The clustering method implemented in admixture assumes that markers are in Hardy–Weinberg (HW) equilibrium, whereas PCA and fineradstructure do not. Loci associated with sex may often fail to be in HW equilibrium, which could thus bias the inference made by admixture.

The two haplotype‐based divergence statistics (i.e., φ_ST_ and *D*_XY_) showed that differences between sexes are linked to scaffold 06, suggesting the presence of genetically differentiated sex chromosomes in *A. mitopteryx* . Homozygosity levels along the genome assembly are similar between the two sexes on all scaffolds except for a major drop in homozygosity in males on scaffold 06, while in females, the homozygosity on scaffold 06 remains within the range of other scaffolds. This observation further supports the presence of differentiated sex chromosomes in males and not in females, and a genetic sex determination system in *A. mitopteryx* , with males being the heterogametic sex.

It is important to note that a chromosome‐level genome assembly for *A. mitopteryx* is not available yet. For this reason, in our study we ordered and oriented scaffolds of the initial *A. mitopteryx* draft assembly into longer pseudoscaffolds according to their order mapping onto the *D. mawsoni* genome assembly. Although *Dissostichus* is supposed to be the most closely related genus to *A. mitopteryx* (Near et al. 2018) and colinearity of genes across notothenioids seems to be well conserved (Bista et al. 2020; Kim et al. 2019; Rayamajhi et al. 2024), it is possible that the highly differentiated sex‐linked markers we recovered, seemingly clustered on a single scaffold, may in fact be distributed across multiple chromosomes or, inversely that other *D. mawsoni* chromosomes, be fused with this scaffold in *A. mitopteryx* . Furthermore, the karyotype of *A. mitopteryx* is still unknown; hence we cannot conclude with certainty that this species has the same number of chromosomes as *Dissostichus* spp. and the hypothetical notothenioid ancestor (2n = 48, Ghigliotti et al. 2007; Pisano et al. 1998), and that *A. mitopteryx* has heteromorphic sex chromosomes. Although XX‐XY is the most common system when males are the heterogametic sex, cytological studies in other notothenioid species described heteromorphic sex chromosomes of either X1X1X2X2‐X1X2Y or XX‐XY1Y2 type in four of the eleven Antarctic notothenioid subfamilies (Nototheniinae, Artedidraconinae, Bathydraconinae, and Channichthyinae) (reviewed in Ghigliotti et al. 2015). Although *D. mawsoni* and *D. eleginoides* do not have heteromorphic sex chromosomes (Ghigliotti et al. 2007), the presence of heteromorphic sex chromosomes in *A. mitopteryx* (such as XY, X1X2Y and XY1Y2) cannot be excluded by our results. The comparison between the genome assemblies of *D. mawsoni* and *C. trigloides* revealed that the sex chromosome in *A. mitopteryx* corresponds to the notothenioid ancestral chromosome 21 (Bista et al. 2020; Kim et al. 2019).

Finally, the absence within the sex‐linked region of known sex‐determining genes or even of a major gene involved in gonad differentiation pathways could be explained if the sex‐determining gene in *A. mitopteryx* is not known to be a sex‐determining gene in other fish species, such as *irf9* in salmonids (Yano et al. 2012) or *idb2* in arapaima ( *Arapaima gigas* , freshwater bonytongue, order Osteoglossiformes; Adolfi, Du, et al. 2021). Also, the fragmentation and incompleteness of the genome assembly in this study could preclude the identification of the sex‐determining gene if located in a part of the genome that was not sequenced and assembled or if it is a duplicated and transposed copy of an autosomal gene, such as in panga catfishes, pikes, or midas cichlids (Kitano et al. 2024; Nacif et al. 2023; Pan, Kay, et al. 2021; Wen et al. 2022), that would have been assembled with its autosomal paralogous gene.

### 4.3 Origin of *A. mitopteryx* Sex Chromosomes

To understand the evolution of the *A. mitopteryx* male heterogametic sex determination system, we reanalyzed a RAD‐seq dataset of about the same number of individuals from *D. mawsoni* , one of the two *Dissotichus* spp. sister species of *A. mitopteryx* . In stark contrast to *A. mitopteryx* , for which even a small number of specimens were sufficient to reveal the presence of differentiated sex chromosomes, we found no evidence of sex‐linked regions in *D. mawsoni* . This result suggests that either the *A. mitopteryx* sex determination system is not shared with *D. mawsoni* , that the *D. mawsoni* sex chromosome is far less differentiated so that it cannot be detected by a RAD‐seq approach, or that the *D. mawsoni* dataset and its sample size were insufficient to detect sex‐linked loci.

The haplotype‐based divergence statistics φ_ST_ and *D*_XY_ vary in the difference of their respective values between scaffold 06 and the rest of the assembly. While the average φ_ST_ calculated from scaffold 06 data is clearly higher than the genome‐wide average, *D*_XY_ values for scaffold 06 are only slightly different from the rest of the genome assembly. Since φ_ST_ is based on haplotypic frequencies and *D*_XY_ relies on the different number of substitutions between groups, it is possible that the differences in the haplotype‐based divergence statistics are due to only a moderate difference between males and females at scaffold 06. Higher values of φ_ST_ compared to *D*_XY_ can also suggest a recent divergence of the putative sex chromosomes because *D*_XY_ tends to reflect divergence on distant time scales while φ_ST_ tends to reflect divergence on a shorter time scale as allele frequencies can change faster than mutations arise (Cruickshank and Hahn 2014). Further, the failure to detect any major differences in sequencing depth between males and females on scaffold 06 suggests little divergence between the sex‐linked regions and thus supports the hypothesis of young sex chromosomes. A chromosome‐level genome assembly for *A. mitopteryx* and a greater depth of sequencing of multiple samples could help test this hypothesis.

Together, both the differentiation of the putative sex chromosomes in *A. mitopteryx* and our inability to detect a similar sex determination system in *D. mawsoni* suggest that the sex determination system of *A. mitopteryx* is species‐specific and that it evolved after the divergence of *A. mitopteryx* and the *Dissostichus* spp. lineages. The alternative hypothesis, that the *A. mitopteryx* sex‐determination system is ancestral and was subsequently lost in the *Dissostichus* lineage, cannot, however, be ruled out because, outside of *Eleginops maclovinus* being the only known hermaphrodite notothenioid (Brickle et al. 2005), the genetic sex determination system in more basally diverging notothenioid species remains to be studied.

## 5 Conclusions

Our study found a lack of spatial and temporal population structure for the pelagic notothenioid species *A. mitopteryx* between the Eastern Antarctic Peninsula and Eastern Weddell Sea. This finding may be explained by oceanic current regimes facilitating passive transport of larvae given the pelagic behavior of adults. Expanding the spatial scale and sample size would help overcome the limitations of this study and enable testing of the hypothesis of large‐scale connectivity in this fish with high potential for dispersal and circum‐Antarctic distribution. The rarity of the species and the difficult logistics of sampling in the Southern Ocean drive the need for a continued monitoring of species connectivity. This implies that more effort is needed to coordinate sampling across different institutes and countries that operate in the Southern Ocean (e.g., Jones et al. 2024). Continuous monitoring of species connectivity will also be increasingly important to track possible range shifts in the Southern Ocean. In the near future, connectivity between the Antarctic Peninsula and the Weddell Sea, which is facilitated by the circulation pattern in the Southern Ocean, might be of crucial importance for cold‐adapted species living in Antarctic waters. The Antarctic Peninsula is one of the polar regions most affected by climate change and human activities, while the Weddell Sea may respond more slowly than other areas of the Southern Ocean to global warming (Raphael et al. 2025; Strass et al. 2020; Teschke et al. 2021; Turner et al. 2020) and has been speculated to act as a sink for migrating cold‐adapted species (Griffiths et al. 2017). For this reason, connectivity between the Antarctic Peninsula and the Weddell Sea is a crucial aspect to monitor, and *A. mitopteryx* can serve as a proxy for pelagic species.

Furthermore, we demonstrated the genetic differentiation between male and female *A. mitopteryx* . We propose that the species has a male heterogametic genetic sex determination system that is located on the notothenioid ancestral chromosome 21 (i.e., scaffold 06 in *D. mawsoni* and *A. mitopteryx* ), even though we failed to identify the exact sex‐determining gene. The finding of sex‐linked markers in *A. mitopteryx* leads to practical and theoretical considerations. Sex‐linked markers may bias estimates of population genetic diversity and differentiation if not properly recognized. Future efforts might target the expansion of robust genetic resources for *A. mitopteryx* to support the identification of sex‐linked markers to develop simple and cheap molecular tests to assign sex to individuals of unknown sex. These resources would valuably aid various types of experimental studies, as well as the description of sex‐specific ecological and evolutionary phenomena without having to sacrifice the individual.

## Author Contributions

**Luca Schiavon:** conceptualization (equal), data curation (equal), formal analysis (equal), methodology (equal), visualization (equal), writing – original draft (equal), writing – review and editing (equal). **Thomas Desvignes:** conceptualization (supporting), formal analysis (supporting), methodology (supporting), validation (supporting), visualization (supporting), writing – original draft (equal), writing – review and editing (equal). **Fabrizia Ronco:** formal analysis (supporting), methodology (supporting), validation (supporting), writing – original draft (supporting), writing – review and editing (equal). **Michael Matschiner:** conceptualization (supporting), formal analysis (supporting), funding acquisition (supporting), methodology (supporting), validation (supporting), writing – original draft (supporting), writing – review and editing (equal). **Martina Gastaldi:** formal analysis (supporting), writing – original draft (supporting), writing – review and editing (supporting). **Thore Koppetsch:** formal analysis (supporting), methodology (supporting), writing – original draft (supporting), writing – review and editing (supporting). **Audun Schrøder‐Nielsen:** formal analysis (supporting), methodology (supporting), writing – original draft (supporting), writing – review and editing (supporting). **John H. Postlethwait:** formal analysis (supporting), methodology (supporting), writing – original draft (supporting), writing – review and editing (supporting). **Alessia Prestanti:** formal analysis (supporting), methodology (supporting), writing – review and editing (supporting). **Federica Stranci:** formal analysis (supporting), methodology (supporting), writing – review and editing (supporting). **Santiago G. Ceballos:** formal analysis (supporting), methodology (supporting), writing – review and editing (supporting). **Felix C. Mark:** investigation (supporting), resources (equal), writing – review and editing (supporting). **Magnus Lucassen:** funding acquisition (supporting), investigation (supporting), resources (equal), writing – review and editing (supporting). **Emilio Riginella:** investigation (supporting), resources (supporting), writing – review and editing (supporting). **Mario La Mesa:** conceptualization (supporting), funding acquisition (equal), investigation (supporting), project administration (equal), writing – original draft (supporting), writing – review and editing (supporting). **Chiara Papetti:** conceptualization (equal), formal analysis (supporting), funding acquisition (equal), investigation (equal), methodology (supporting), project administration (lead), resources (equal), supervision (lead), writing – original draft (equal), writing – review and editing (equal).

## Ethics Statement

Fish were sampled and processed according to and within the laws, guidelines, and policies of the German and European Animal Welfare legislations. Sample collection during the cruises with R/V *Polarstern* was approved by the competent German authority for Antarctic research, the UBA (*Umweltbundesamt*).

## Conflicts of Interest

The authors declare no conflicts of interest.

## Supporting information


Data S1.

## Data Availability

All the codes, datasets and accessory files needed to replicate the analyses are available at https://github.com/Papetti‐Lab/Aethotaxis_RAD‐Seq. Raw sequence reads are available under the NCBI BioProject Accession number: PRJNA1214794 (https://www.ncbi.nlm.nih.gov/sra/PRJNA1214794).
