# Supplementary material for: Sex Differentiation and Long‐Distance Gene Flow in the Elusive Antarctic Fish Aethotaxis mitopteryx
Source: Ecol Evol. 2025 Aug 20;15(8):e71847. doi: 10.1002/ece3.71847 (PMC12367274; doi:10.1002/ece3.71847)
Supplement: Supplementary file 1 — Data S1. [file ECE3-15-e71847-s001.docx]

**Supplemental Information for:**

**Sex differentiation and long-distance gene flow in the elusive Antarctic fish *Aethotaxis mitopteryx***

Luca Schiavon^1^, Thomas Desvignes^2,3^, Fabrizia Ronco^4,5^, Michael Matschiner^4^, Martina Gastaldi^1^, Thore Koppetsch^4^, Audun Schrøder-Nielsen^4^, John H. Postlethwait^2^, Alessia Prestanti^1,4^, Federica Stranci^1^, Santiago G. Ceballos^6^, Felix C. Mark^7^, Magnus Lucassen^7^, Emilio Riginella^8^, Mario La Mesa^9^, Chiara Papetti^1,10*^

1 Biology Department, University of Padova, Padova, Italy

2 Institute of Neuroscience, University of Oregon, Eugene, Oregon, USA

3 Department of Biology, University of Alabama at Birmingham, Birmingham, Alabama, USA

4 Natural History Museum, University of Oslo, Oslo, Norway

5 Department of Environmental Sciences, University of Basel, Basel, Switzerland

6 Instituto de Ciencias Polares, Ambiente y Recursos Naturales (ICPA), Universidad Nacional de Tierra del Fuego (UNTDF), Ushuaia, Argentina

7 Alfred Wegener Institute Helmholtz Centre for Polar and Marine Research, Bremerhaven, Germany

8 Zoological Station Anton Dohrn, Napoli, Italy

9 CNR‑ISP, Institute of Polar Sciences, c/o Area della Ricerca di Bologna, Bologna, Italy

10 National Biodiversity Future Centre, Palermo, Italy

**Figure S1:** Graph of the cross-validation results of Admixture analysis.


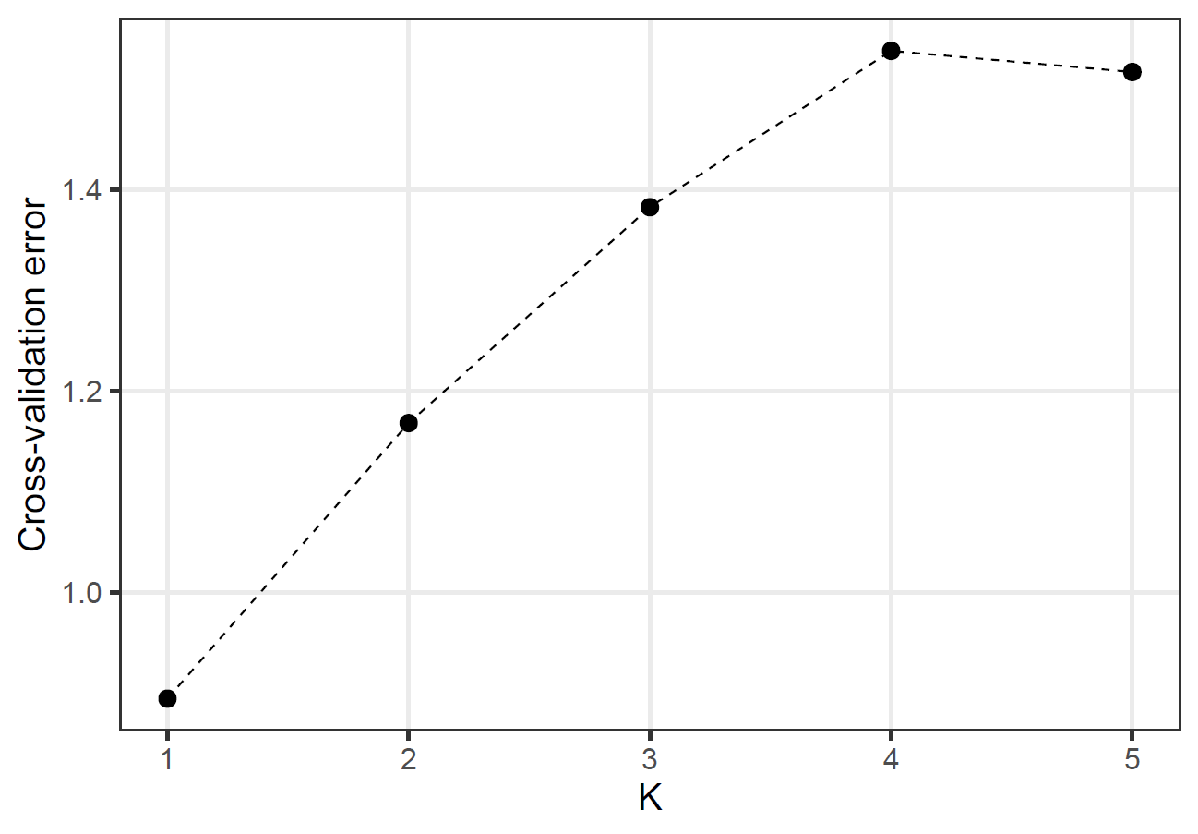


**Figure S2:** Admixture plot representing individual membership probabilities considering two clusters (*K* = 2) as indicated by Admixture. Each individual is represented by a vertical lane, the proportion of different gray-scales in each lane is proportional to the probability of assignment to each cluster. Pink circles represent females, blue squares represent males. The plot on the top shows clustering results based on the entire SNP dataset, the bottom plot is based on a dataset excluding SNPs mapped on scaffold 06 (the scaffold that shows sex differences).


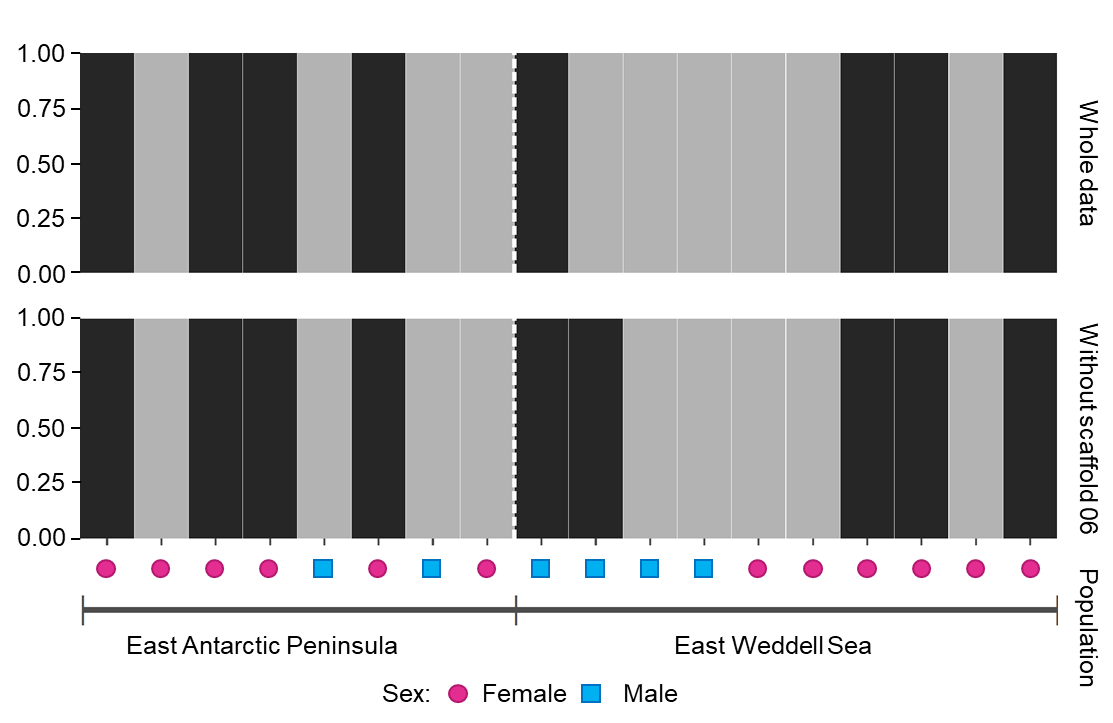


**Figure S3:** Co-ancestry matrix inferred by fineRADstructure. For each row and for each column, every individual is compared to all other specimens. Pink circles represent females, blue squares represent males. Co-ancestry ranges from weak (yellow) to strong (blue) as indicated by the colour scale. In the matrix, dark blue and purple indicate individuals with larger proportions of loci with shared co-ancestry than others. Black dashed lines delimit the two sexes.


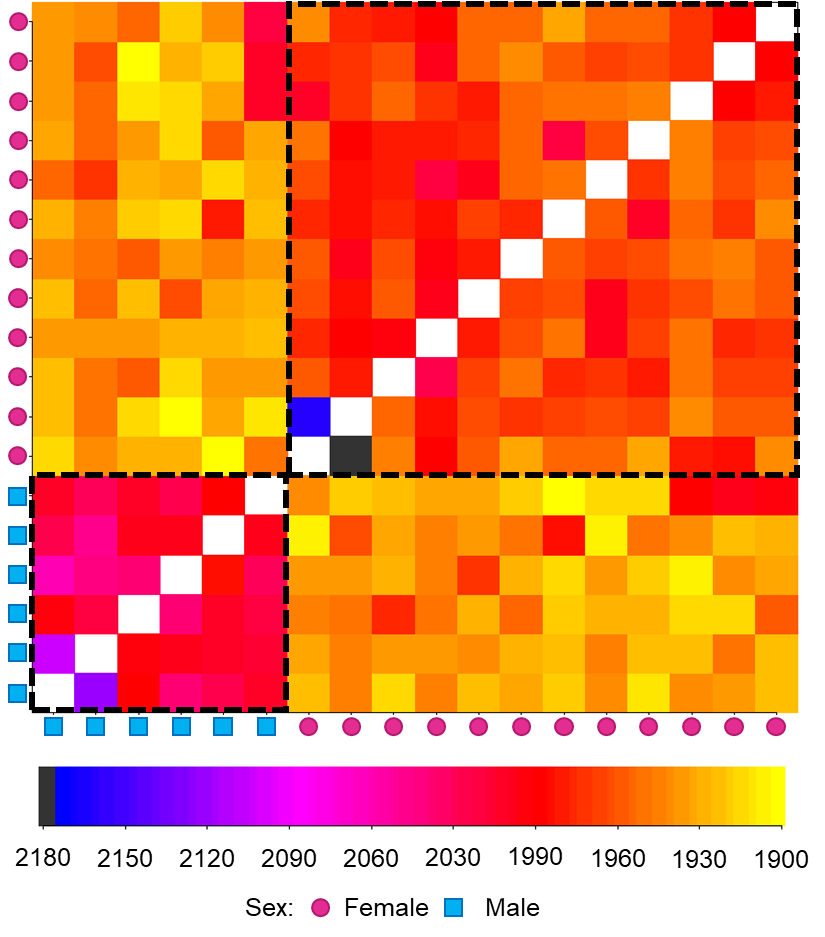


**Figure S4:** Manhattan plot of haplotype-based divergence statistics φ_ST_ (panel A) and D_XY_ (panel B) calculated between Eastern Antarctic Peninsula and the Eastern Weddell Sea specimens. Alternating black and grey dots represent single site values in different scaffolds. φ_ST_ smoothed average is displayed in green, D_XY_ smoothed average is displayed in purple.


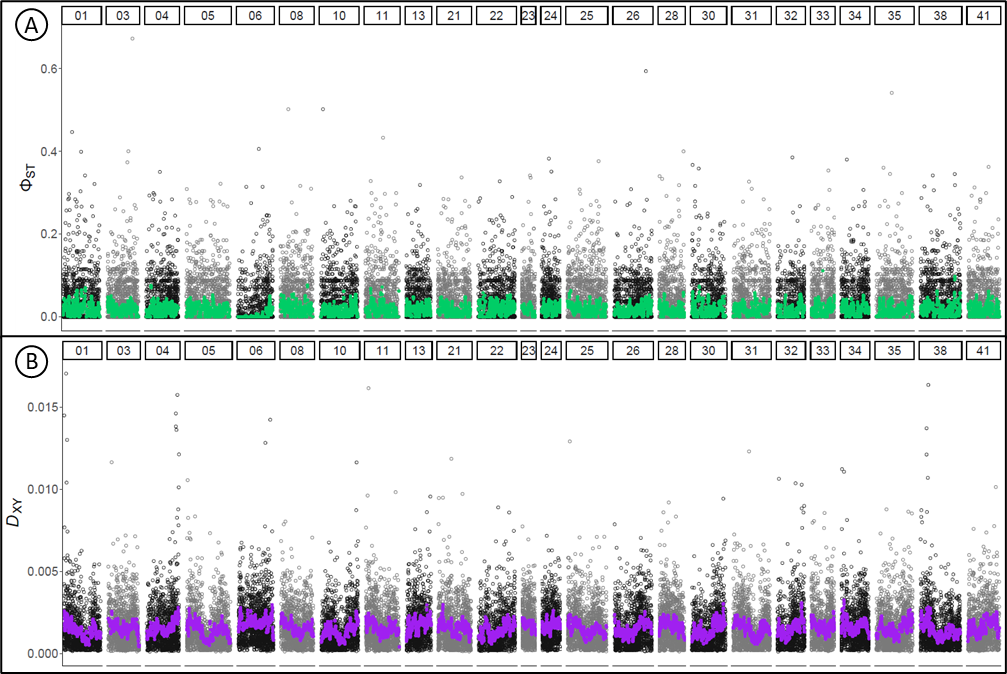


**Figure S5:** Scatter plot of PCA excluding SNPs mapped on scaffold 06 (the scaffold that shows sex differences). Labels on the axes indicate the contributions of the displayed principal components to the variance.


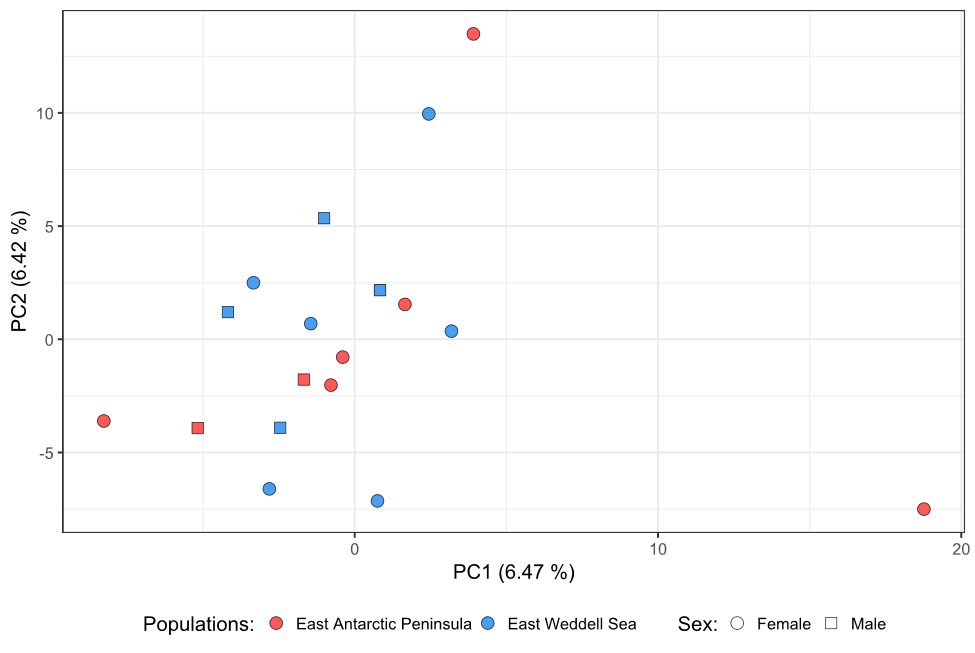


**Figure S6:** Co-ancestry matrix inferred by fineRADstructure excluding data from scaffold 06 (the scaffold that shows sex differences). For each row and for each column, every individual is compared to all other specimens. Pink circles represent females, blue squares represent males. Co-ancestry ranges from weak (yellow) to strong (blue) as indicated by the colour scale. In the matrix, dark blue and purple indicate individuals with larger proportions of loci with shared co-ancestry than others.


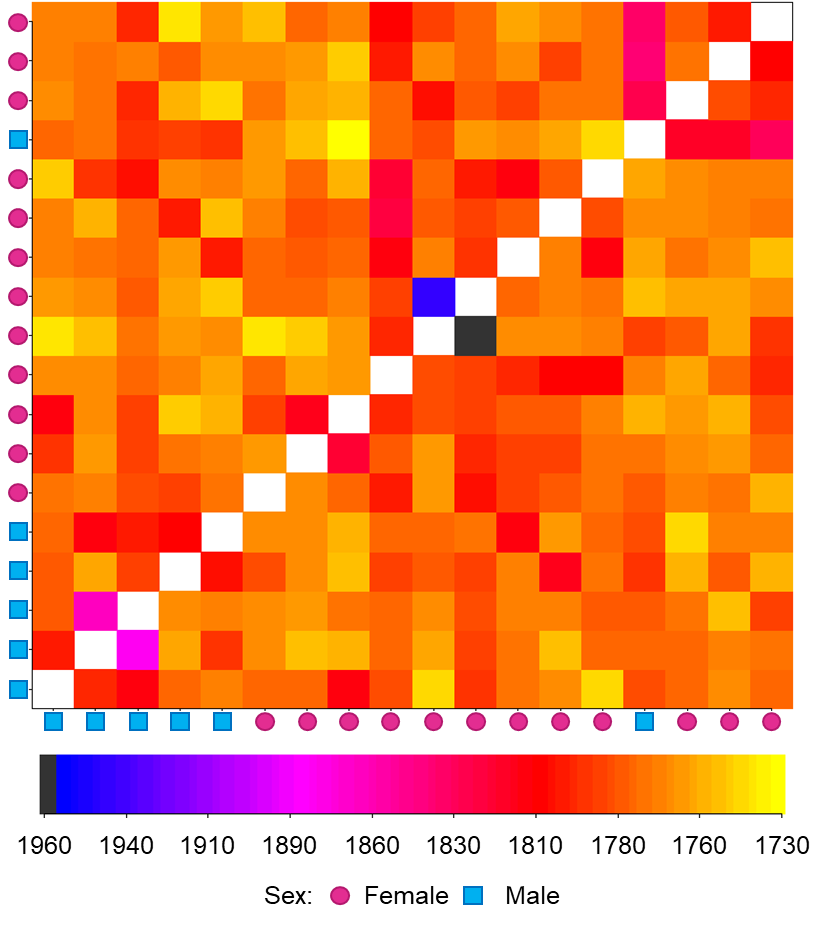


**Figure S7:** Linkage disequilibrium between SNPs along separate scaffolds, males and females are indicated by different colours.
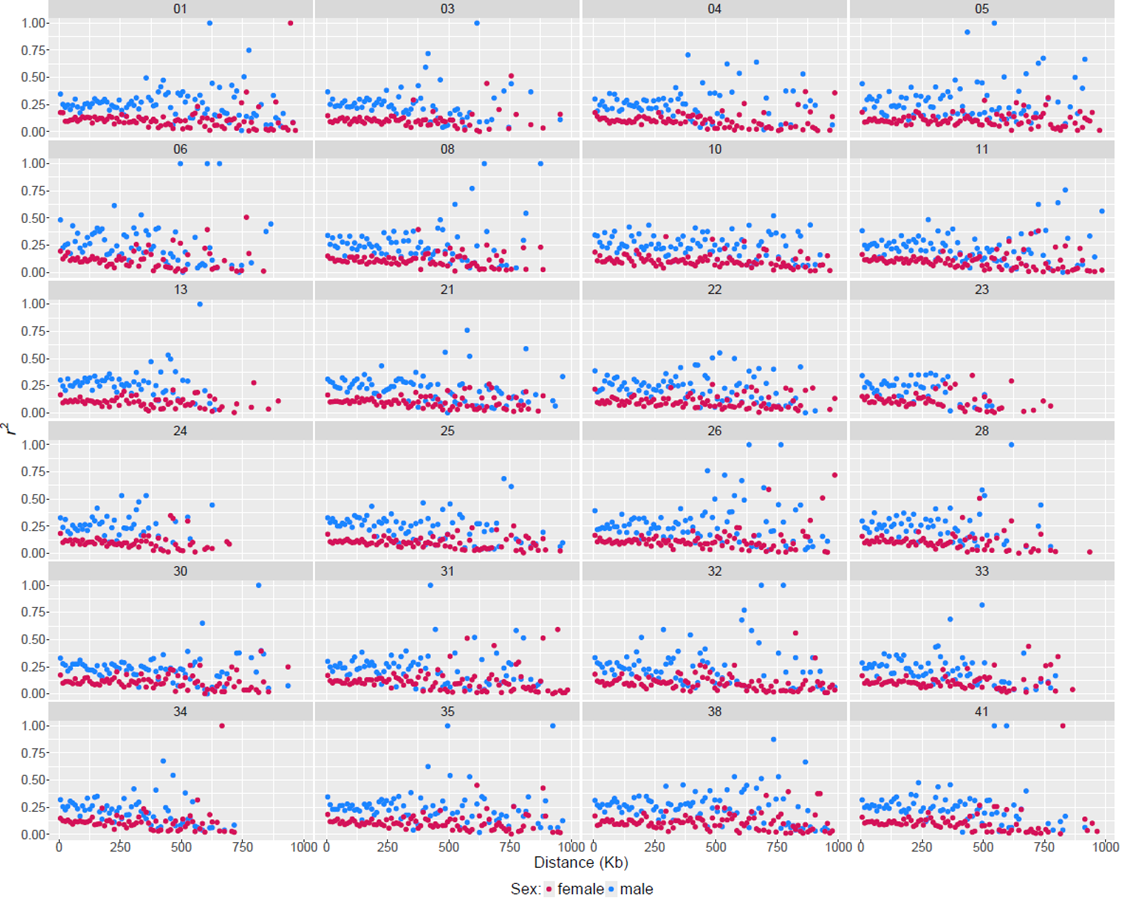


**Figure S8:** Tile plots produced by RADsex representing the distribution of *Aethotaxis mitopteryx* markers between males and females, based on different thresholds for minimum depth adopted during the analysis. The colour of each tile represents the number of markers associated with the corresponding number of males and females indicated respectively in the horizontal and vertical axis. The darker the tile, the higher the number of markers in males (x axis) or females (y axis). Male-biased markers are located in the bottom-right corner of the plots while female-biased markers are located in the top-left corner of the plots.


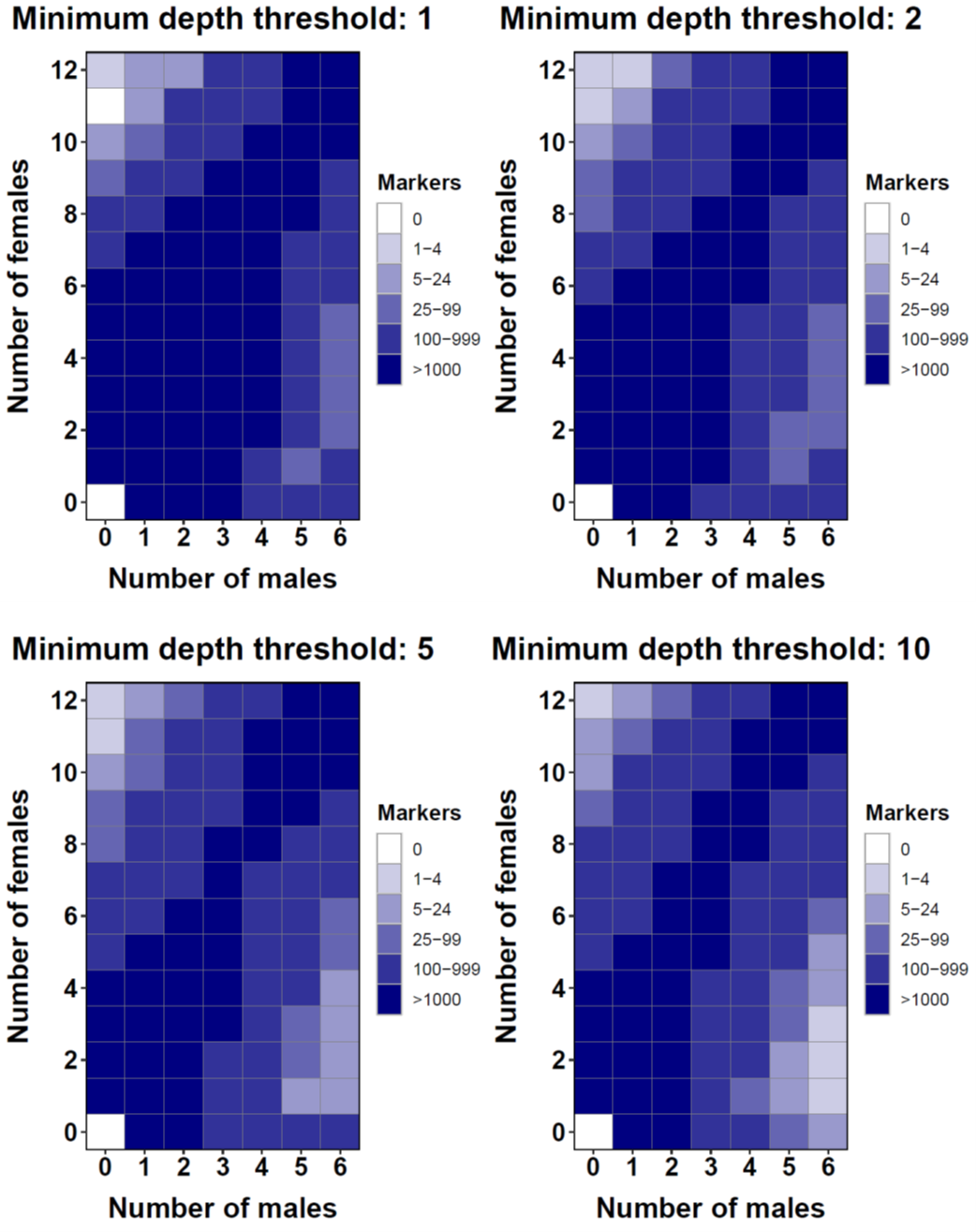


**Figure S9:** Tile plots produced by RADsex indicating the distribution of *Aethotaxis mitopteryx* markers between males and females, considering using only the markers on Scaffold 06 and at different sequencing depth thresholds. The colour of each tile represents the number of markers associated with the corresponding number of males and females indicated respectively in the horizontal and vertical axis. The darker the tile the higher the number of markers in males (x axis) or females (y axis). Male-biased markers are located in the bottom-right corner of the plots while female-biased markers are located in the top-left corner of the plots.


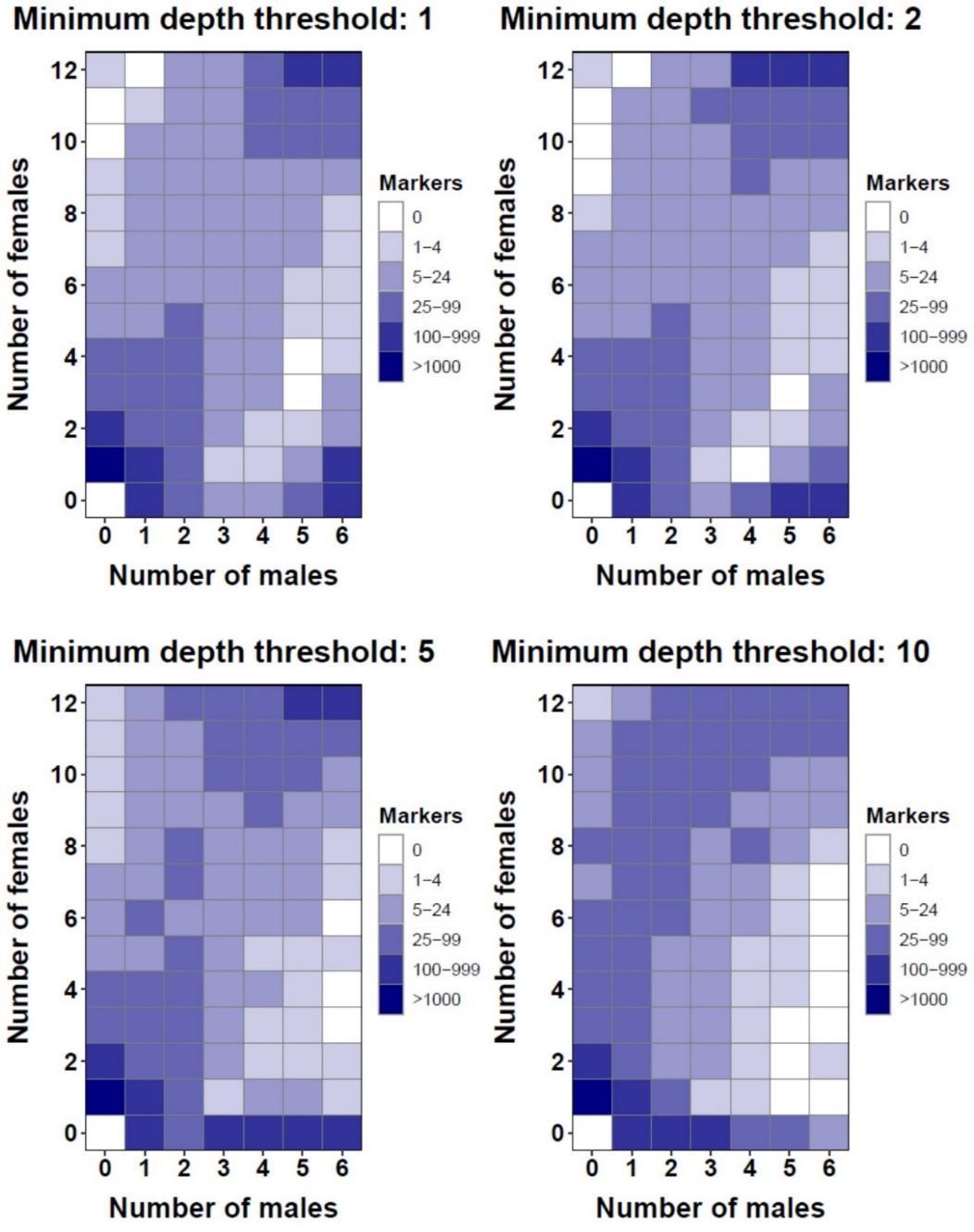


**Figure S10:** Dot-plot visualisation of the alignment of *Dissostichus mawsoni* scaffold 06 against *Cottoperca trigloides* chromosome 21. The scale on each axis is in megabases (Mb). The points represent the similarity between the two sequences (the correspondence between colours and similarity values is shown in the inset). The dots along the diagonal represent colinear genomic regions whereas the dots off the diagonal represent rearrangements (insertions, deletions, inversions and translocations). Overall, the plot shows congruence between the aligned sequences, indicating that *D. mawsoni* scaffold 06 corresponds to *C. trigloides* chromosome 21.


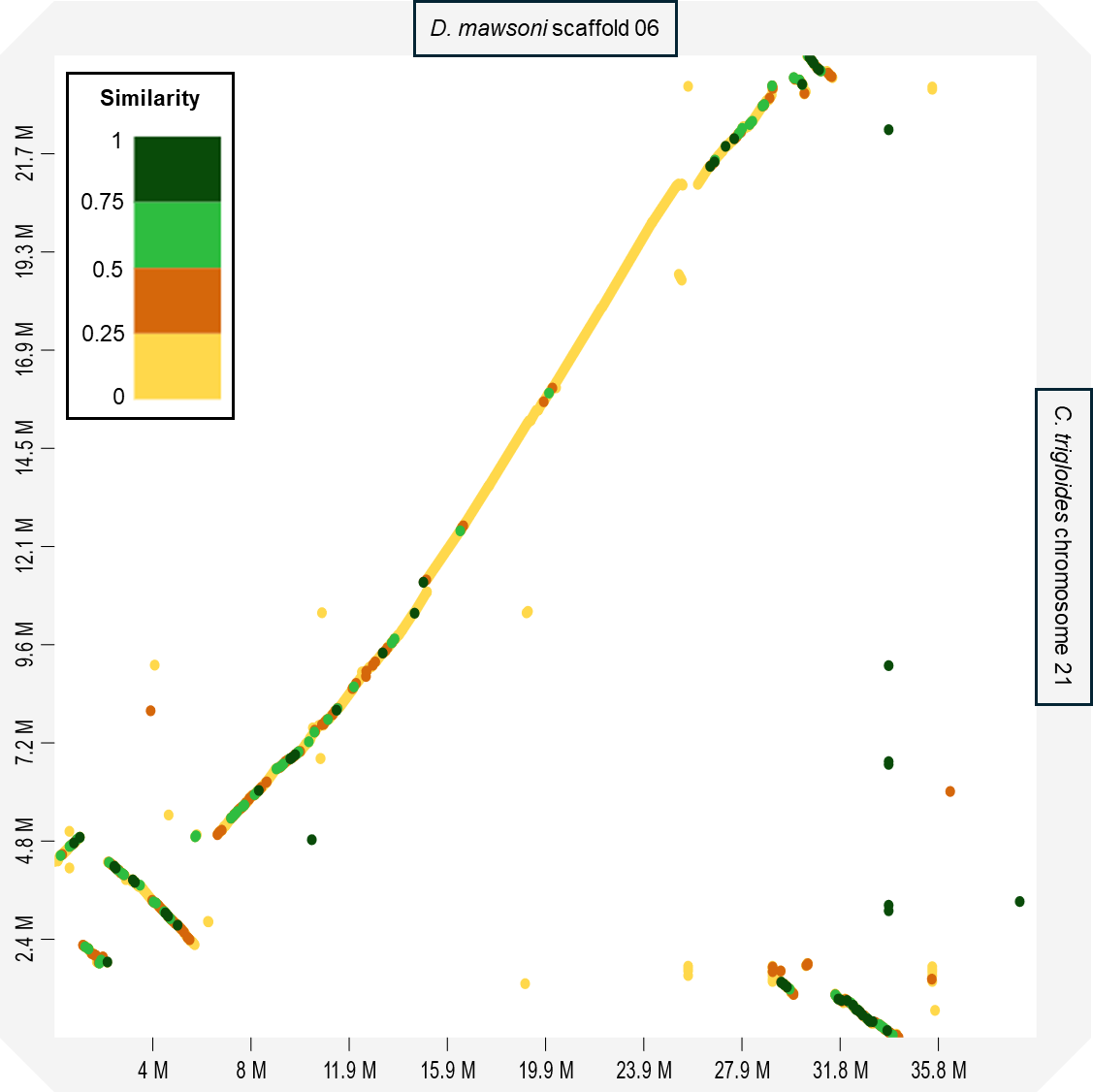


**Figure S11:** Tile plots produced by RADsex indicating the distribution of *D. mawsoni* markers between males and females, considering different thresholds for minimum sequencing depth adopted during the analysis. The colour of each tile represents the number of markers associated with the corresponding number of males and females indicated respectively in the horizontal and vertical axis. The darker the tile the higher the number of markers in males (x axis) or females (y axis). Male-biased markers are located in the bottom-right corner of the plots while female-biased markers are located in the top-left corner of the plots.


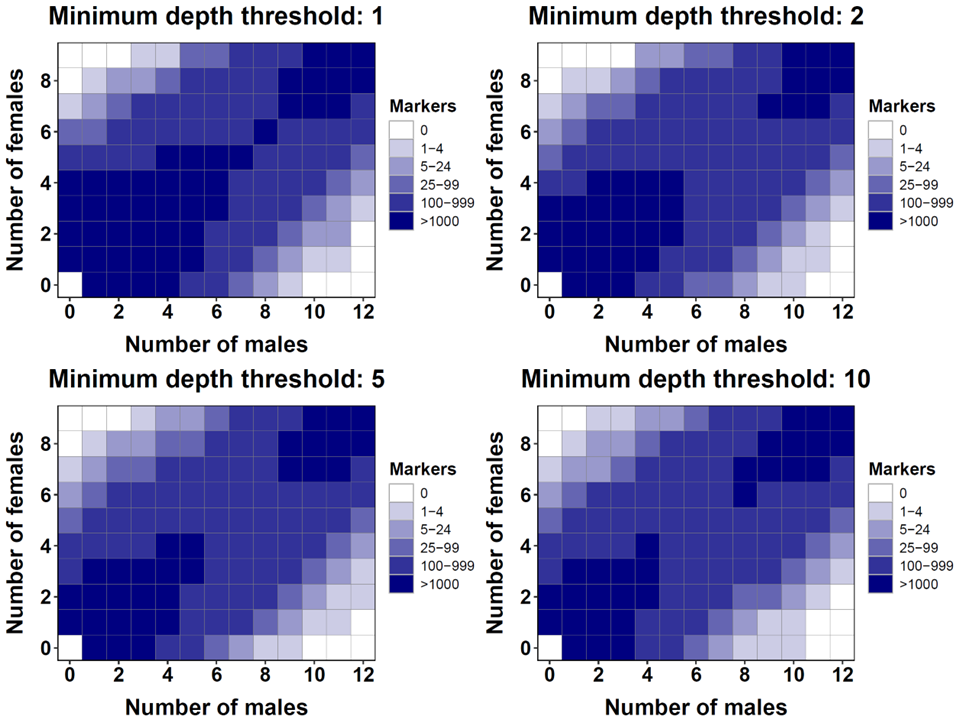


**Figure S12:** Plot showing the distribution of markers between males and females of *Dissostichus mawsoni*, considering a sequencing depth threshold of 10. Each sector represents a different scaffold. The top track indicates the probability of association with sex. The bottom track indicates the bias of a marker: if a marker is present in all males and no females, it has a value of 1; if a marker is present in all females and no males, it has a value of -1.


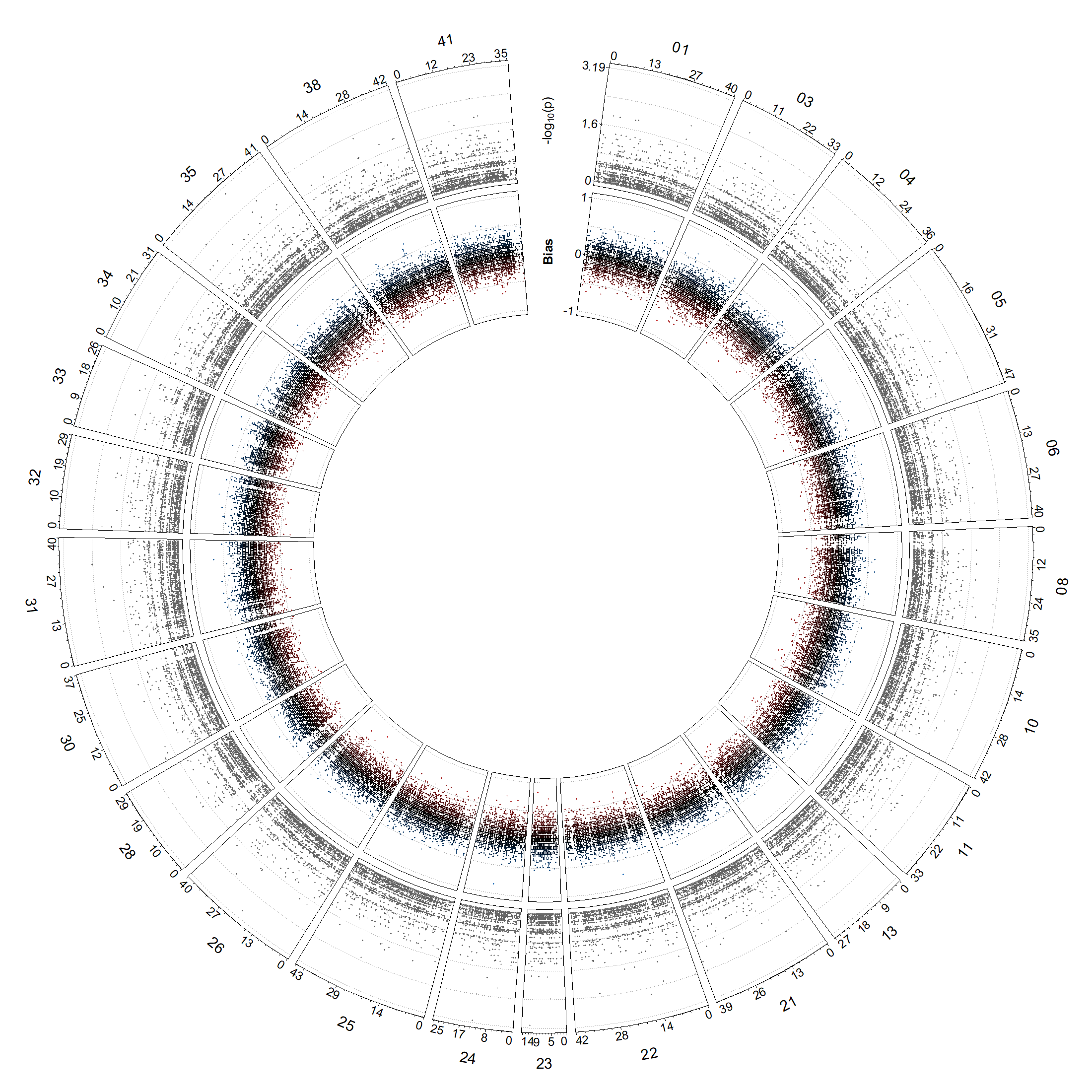


**Table S1:** Table of the candidate sex determining genes in the *Aethotaxis mitopteryx* genome assembly. The table contains gene names, NCBI reference sequence accession numbers and reference species used for the BLAST search (*Trematomus bernachii*, *Perca flavescens*). The scaffold of the best BLAST hit on the *A. mitopteryx* assembly is reported for all genes. When the best hit was on scaffolds in which significant RADsex markers were found, the alignment start, end, and orientation of the hit are provided along with the position of the closest male-linked marker and the distance between the marker and the gene hit. If no significant RADsex markers were found on the best hit’s scaffold, the alignment position was not provided. Chr0 indicates the pseudoscaffold that contains all the unplaced contigs.

| **Gene** | **Species** | **Accession number** | **Best hit on *A. mitopteryx* Assembly** | **Gene Start** | **Gene End** | **Strand** | **Closest male-linked marker** | **Distance to closest marker** | **Notes** |
| --- | --- | --- | --- | --- | --- | --- | --- | --- | --- |
| *amh* | *T. bernachii* | XM_034146407.1 | 38 | N/A | N/A | N/A | N/A | N/A | N/A |
| *amhr2* | *T. bernachii* | XM_034138719.1 | 25 | N/A | N/A | N/A | N/A | N/A | N/A |
| *bcar1* | *T. bernachii* | XM_034141725.1 | 26 | N/A | N/A | N/A | N/A | N/A | N/A |
| *bmp15* | *T. bernachii* | XM_034145529.1 | Chr0 | N/A | N/A | N/A | N/A | N/A | N/A |
| *bmp4* | *T. bernachii* | XM_034126118.1 | 4 | N/A | N/A | N/A | N/A | N/A | N/A |
| *bmpr1ba* | *T. bernachii* | XM_034117554.1 | 31 | N/A | N/A | N/A | N/A | N/A | N/A |
| *bmpr1bb* | *T. bernachii* | XM_034122669.1 | 34 | 13545595 | 13483339 | - | 17464031 | 3918436 | Only one sex-linked marker on scaffold 34, 3.9Mb away. |
| *bmpr2-like1* | *T. bernachii* | XM_034151117.1 | 23 | N/A | N/A | N/A | N/A | N/A | N/A |
| *bmpr2-like2* | *T. bernachii* | XM_034148480.1 | 6 | 26150998 | 26131708 | - | 24370159 | 1761549 | 1.8Mb away from the last sex-linked marker and 6.9Mb away from second to last. |
| *ctnnb1* | *T. bernachii* | XM_034127418.1 | 1 | N/A | N/A | N/A | N/A | N/A | N/A |
| *cyp19a1* | *T. bernachii* | XM_034116796.1 | 5 | N/A | N/A | N/A | N/A | N/A | N/A |
| *dmrt1* | *T. bernachii* | XM_034118725.1 | 31 | N/A | N/A | N/A | N/A | N/A | N/A |

**Table S1 (continuation):**

| **Gene** | **Species** | **Accession number** | **Best hit on *A. mitopteryx* Assembly** | **Gene Start** | **Gene End** | **Strand** | **Closest male-linked marker** | **Distance to closest marker** | **Notes** |
| --- | --- | --- | --- | --- | --- | --- | --- | --- | --- |
| *esr1* | *T. bernachii* | XM_034142804.1 | 32 | 11537296 | 11548810 | + | 2400301 | 9136995 | Only one sex-linked marker on scaffold 32, 9.1Mb away. |
| *fgf24* | *T. bernachii* | XM_034133830.1 | 22 | N/A | N/A | N/A | N/A | N/A | N/A |
| *foxl2a* | *T. bernachii* | XM_034114634.1 | 35 | N/A | N/A | N/A | N/A | N/A | N/A |
| *foxl2l* | *T. bernachii* | XM_034145116.1 | 10 | N/A | N/A | N/A | N/A | N/A | N/A |
| *fshr* | *T. bernachii* | XM_034119560.1 | 28 | N/A | N/A | N/A | N/A | N/A | N/A |
| *fsta* | *T. bernachii* | XM_034118622.1 | 1091 | N/A | N/A | N/A | N/A | N/A | N/A |
| *gata4* | *T. bernachii* | XM_034142247.1 | 32 | 9760896 | 9768924 | + | 2400301 | 7360595 | Only one sex-linked marker on scaffold 32, 7.4Mb away. |
| *gdf6a* | *T. bernachii* | XM_034127829.1 | 1 | N/A | N/A | N/A | N/A | N/A | N/A |
| *gdf6b* | *T. bernachii* | XM_034125362.1 | 3 | N/A | N/A | N/A | N/A | N/A | N/A |
| *gdf9* | *T. bernachii* | XM_034121319.1 | 22 | N/A | N/A | N/A | N/A | N/A | N/A |
| *gsdf* | *T. bernachii* | XM_034123582.1 | 34 | 14541440 | 14543702 | + | 17464031 | 2922591 | Only one sex-linked marker on scaffold 34, 2.9Mb away. |
| *hsd17b1* | *T. bernachii* | XM_034143819.1 | 41 | N/A | N/A | N/A | N/A | N/A | N/A |
| *hsd17b3* | *T. bernachii* | XM_034118667.1 | 31 | N/A | N/A | N/A | N/A | N/A | N/A |
| *id2a* | *T. bernachii* | XM_034124719.1 | 4 | N/A | N/A | N/A | N/A | N/A | N/A |
| *irf9* | *T. bernachii* | XM_034146681.1 | 33 | N/A | N/A | N/A | N/A | N/A | N/A |
| *nr5a1a* | *P. flavescens* | XM_028577969.1 | 31 | N/A | N/A | N/A | N/A | N/A | N/A |
| *rspo1* | *T. bernachii* | XM_034153344.1 | 1 | N/A | N/A | N/A | N/A | N/A | N/A |
| *sox2* | *T. bernachii* | XM_034147755.1 | 38 | N/A | N/A | N/A | N/A | N/A | N/A |
| *sox3* | *T. bernachii* | XM_034138356.1 | 22 | N/A | N/A | N/A | N/A | N/A | N/A |

**Table S1 (continuation):**

| **Gene** | **Species** | **Accession number** | **Best hit on *A. mitopteryx* Assembly** | **Gene Start** | **Gene End** | **Strand** | **Closest male-linked marker** | **Distance to closest marker** | **Notes** |
| --- | --- | --- | --- | --- | --- | --- | --- | --- | --- |
| *sox9a* | *T. bernachii* | XM_034143142.1 | 41 | N/A | N/A | N/A | N/A | N/A | N/A |
| *sult1st6* | *T. bernachii* | XM_034119228.1 | 28 | N/A | N/A | N/A | N/A | N/A | N/A |
| *wnt4a* | *T. bernachii* | XM_034121966.1 | 25 | N/A | N/A | N/A | N/A | N/A | N/A |
| *wnt4b* | *T. bernachii* | XM_034145761.1 | 1 | N/A | N/A | N/A | N/A | N/A | N/A |

**Table S2:** Quality and completeness statistics are provided for three stages of the *A. mitopteryx* genome assembly: (i) after the initial short-read-only assembly, (ii) after the incorporation of low-coverage long-reads, which is the version used in this study prior to *D. mawsoni*-guided scaffolding, and (iii) the final version submitted to NCBI after the decontamination step.

| **Assembly version** | **Short-reads only assembly** | **Used in this study (short and long reads)** | **Submitted to Genbank** |
| --- | --- | --- | --- |
| **n° contigs (length >= 0 bp)** | 2,832,590 | 2,817,267 | 1,032,909 |
| **n° contigs (>= 1000 bp)** | 89,089 | 77,608 | 77,593 |
| **n° contigs (>= 5000 bp)** | 27,882 | 27,396 | 27,394 |
| **n° contigs (>= 10000 bp)** | 11,901 | 13,649 | 13,648 |
| **n° contigs (>= 25000 bp)** | 2,225 | 3380 | 3,380 |
| **n° contigs (>= 50000 bp)** | 328 | 657 | 657 |
| **Total length (>= 0 bp)** | 1,066,530,475 | 1,084,446,515 | 833,399,207 |
| **Total length (>= 1000 bp)** | 486,828,563 | 506,935,727 | 506,884,282 |
| **Total length (>= 5000 bp)** | 340,056,647 | 390,639,245 | 390,617,219 |
| **Total length (>= 10000 bp)** | 228,256,921 | 294,023,595 | 294,006,947 |
| **Total length (>= 25000 bp)** | 83,592,805 | 137,009,863 | 137,009,863 |
| **Total length (>= 50000 bp)** | 21,445,146 | 45,671,112 | 45,671,112 |
| **n° contigs** | 225,225 | 210,180 | 209,966 |
| **Largest contig** | 193,310 | 193,562 | 193,562 |
| **Total length** | 578,601,181 | 595,942,291 | 595,770,866 |
| **GC (%)** | 40.07 | 40.11 | 40.11 |
| **N50** | 6,965 | 9740 | 9,746 |
| **L50** | 19,256 | 14,049 | 14,041 |
| **n° of Ns per 100 kbp** | 480.14 | 1,357.59 | 1,357.83 |
| **Actinopterygii BUSCO:**  **complete (%)** | 55.5 | 61.3 | 61.3 |
| **Actinopterygii BUSCO:**  **complete & single copy (%)** | 53.8 | 59.3 | 59.3 |
| **Actinopterygii BUSCO:**  **complete & duplicated (%)** | 1.7 | 2 | 2 |
| **Actinopterygii BUSCO: fragmented (%)** | 13.5 | 12.4 | 12.4 |
| **Actinopterygii genes:**  **missing BUSCO (%)** | 31.0 | 26.3 | 26.3 |

**Table S3:** Absolute numbers and percentage of contigs and base pairs of the *A. mitopteryx* assembly that were placed into pseudoscaffolds with the software RagTag using as a guide the assembly of *D. mawsoni*.

| **n° original sequences** | 2,817,267 |
| --- | --- |
| **original total length** | 1,084,446,515 |
| **n° placed sequences** | 297,446 |
| **percentage placed sequences** | 10.6 |
| **n° placed base pairs** | 559,655,217 |
| **percentage placed base pairs** | 51.6 |
| **n° unplaced sequences** | 2,519,821 |
| **percentage placed base pairs** | 89.4 |
| **n° unplaced bp** | 524,791,298 |
| **percentage unplaced base pairs** | 48.4 |

**Table S4:** Number and length of the scaffolds of the *A. mitopteryx* assembly after it was assembled into pseudochromosomes with RagTag. The higher length of this version compared to the original *A. mitopteryx* assembly is due to the introduction of gaps during the alignment with the *D. mawsoni* assembly.

| **n° scaffolds** | 311 |
| --- | --- |
| **total length** | 1,366,142,115 |
| **scaffold minimum length** | 98 |
| **scaffold average length** | 4,392,739.90 |
| **scaffold maximum length** | 776,773,298 |
